# Comprehensive analyses of partially methylated domains and differentially methylated regions in esophageal cancer reveal both cell-type- and cancer-specific epigenetic regulation

**DOI:** 10.1186/s13059-023-03035-3

**Published:** 2023-08-24

**Authors:** Yueyuan Zheng, Benjamin Ziman, Allen S. Ho, Uttam K. Sinha, Li-Yan Xu, En-Min Li, H Phillip Koeffler, Benjamin P. Berman, De-Chen Lin

**Affiliations:** 1 Clinical Big Data Research Center, Scientific Research Center, The Seventh Affiliated Hospital of Sun Yat-Sen University, Shenzhen, 518107 People’s Republic of China; 2 Department of Medicine, Samuel Oschin Comprehensive Cancer Institute, Cedars-Sinai Medical Center, Los Angeles, USA; 3 Center for Craniofacial Molecular Biology, Herman Ostrow School of Dentistry, and Norris Comprehensive Cancer Center, University of Southern California, 2250 Alcazar Street – CSA 207D, Los Angeles, CA 90033 USA; 4 Division of Otolaryngology-Head and Neck Surgery, Department of Surgery, Samuel Oschin Cancer Center, Cedars-Sinai Medical Center, Los Angeles, CA USA; 5 Department of Otolaryngology, Keck School of Medicine, University of Southern California, Los Angeles, USA; 6 The Key Laboratory of Molecular Biology for High Cancer Incidence Coastal Chaoshan Area, Shantou University Medical College, Guangdong, China; 7 Department of Developmental Biology and Cancer Research, Institute for Medical Research Israel-Canada, Faculty of Medicine, The Hebrew University of Jerusalem, Jerusalem, Israel

**Keywords:** Esophageal cancer, Partially methylated domains, DMRs, Cell-type specificity

## Abstract

**Background:**

As one of the most common malignancies, esophageal cancer has two subtypes, squamous cell carcinoma and adenocarcinoma, arising from distinct cells-of-origin. Distinguishing cell-type-specific molecular features from cancer-specific characteristics is challenging.

**Results:**

We analyze whole-genome bisulfite sequencing data on 45 esophageal tumor and nonmalignant samples from both subtypes. We develop a novel sequence-aware method to identify large partially methylated domains (PMDs), revealing profound heterogeneity at both methylation level and genomic distribution of PMDs across tumor samples. We identify subtype-specific PMDs that are associated with repressive transcription, chromatin B compartments and high somatic mutation rate. While genomic locations of these PMDs are pre-established in normal cells, the degree of loss is significantly higher in tumors. We find that cell-type-specific deposition of H3K36me2 may underlie genomic distribution of PMDs. At a smaller genomic scale, both cell-type- and cancer-specific differentially methylated regions (DMRs) are identified for each subtype. Using binding motif analysis within these DMRs, we show that a cell-type-specific transcription factor HNF4A maintains the binding sites that it generates in normal cells, while establishing new binding sites cooperatively with novel partners such as FOSL1 in esophageal adenocarcinoma. Finally, leveraging pan-tissue single-cell and pan-cancer epigenomic datasets, we demonstrate that a substantial fraction of cell-type-specific PMDs and DMRs identified here in esophageal cancer are actually markers that co-occur in other cancers originating from related cell types.

**Conclusions:**

These findings advance our understanding of DNA methylation dynamics at various genomic scales in normal and malignant states, providing novel mechanistic insights into cell-type- and cancer-specific epigenetic regulations.

**Supplementary Information:**

The online version contains supplementary material available at 10.1186/s13059-023-03035-3.

## Background

Ranking seventh in cancer incidence and sixth in mortality worldwide, esophageal carcinoma is highly aggressive and its patients have poor outcomes, with a 5-year survival rate lower than 20% [1, 2]. Esophageal cancer comprises two major histologic subtypes: squamous cell carcinoma (ESCC) and adenocarcinoma (EAC). These two subtypes have distinct clinical characteristics. ESCC occurs predominantly in the upper and mid-esophagus; EAC is prevalent in the lower esophagus near the gastroesophageal junction (GEJ) and is associated with the precursor lesion known as Barrett’s esophagus (BE). Biologically, ESCC arises from the squamous epithelial cells and has common features with other squamous cell carcinomas (SCC), such as head and neck SCC (HNSCC). In comparison, EAC has columnar cell features and shares many characteristics with tubular gastrointestinal adenocarcinomas. In particular, EAC is almost indistinguishable from GEJ adenocarcinoma in terms of genomic, biological and clinical features.

Epigenetically, multiple studies have reported molecular changes in esophageal cancer, especially at the DNA methylation level [3–9]. For example, methylation differences across thousands of loci between ESCC and EAC were noted by The Cancer Genome Atlas (TCGA) consortium [3]. However, these prior works focused largely on the analyses of DNA methylation in gene promoter regions, which only make up ~ 6% of all CpG sites across the human genome. DNA methylation is known to play important roles in other noncoding regions, such as enhancers [10], partially methylated domains (PMDs) [11], as well as repetitive elements [12]. Therefore, the DNA methylome of esophageal cancer awaits further and comprehensive characterization through genome-wide single-base resolution approaches such as whole-genome bisulfite sequencing (WGBS).

CpG island (CGI) promoter hypermethylation and global DNA hypomethylation are two epigenomic hallmarks in cancer [13]. In most healthy tissues, the vast majority of CpG sites (> 80%) across the genome are fully methylated, except for the CpG-rich regions (e.g., CGIs) and other regulatory elements (predominantly enhancers) [14]. Indeed, focal demethylation is a reliable signature of gene promoters and enhancers, and their methylation levels are robustly maintained across healthy tissues. Additionally, methylation patterns of CpG sites across the genome are notably variable across various normal cell types and can be grouped into cell-type-specific differentially methylated regions (DMRs), which are linked to cell-type-specific regulatory regions [14, 15]. By contrast, abnormal CGI promoter hypermethylation is frequently observed in cancer, which is commonly associated with long-term and stable gene repression [14].

With respect to the global methylation loss, large hypomethylated blocks, also known as PMDs, cover more than one-third of the genome and coincide with heterochromatin, chromatin “B” compartment (determined by Hi-C), and nuclear lamina-associated domains [16–18]. We and others recently found that accumulation of PMD hypomethylation is linked to cumulative mitotic cell divisions, late replication timing, and the deposition of the histone mark H3K36me3 [19, 20]. Functionally, PMDs are associated with inactive gene transcription and heightened genomic instability and may be accompanied by activation of transposable elements (TEs) [19, 21]. While incompletely understood, the majority of the PMD regions are possibly shared across developmental lineages [19]. However, there are enough cell-type specific PMDs to differentiate between different cancer cell types [17, 22, 23] and between different healthy cell types [24].

**Fig. 1:**
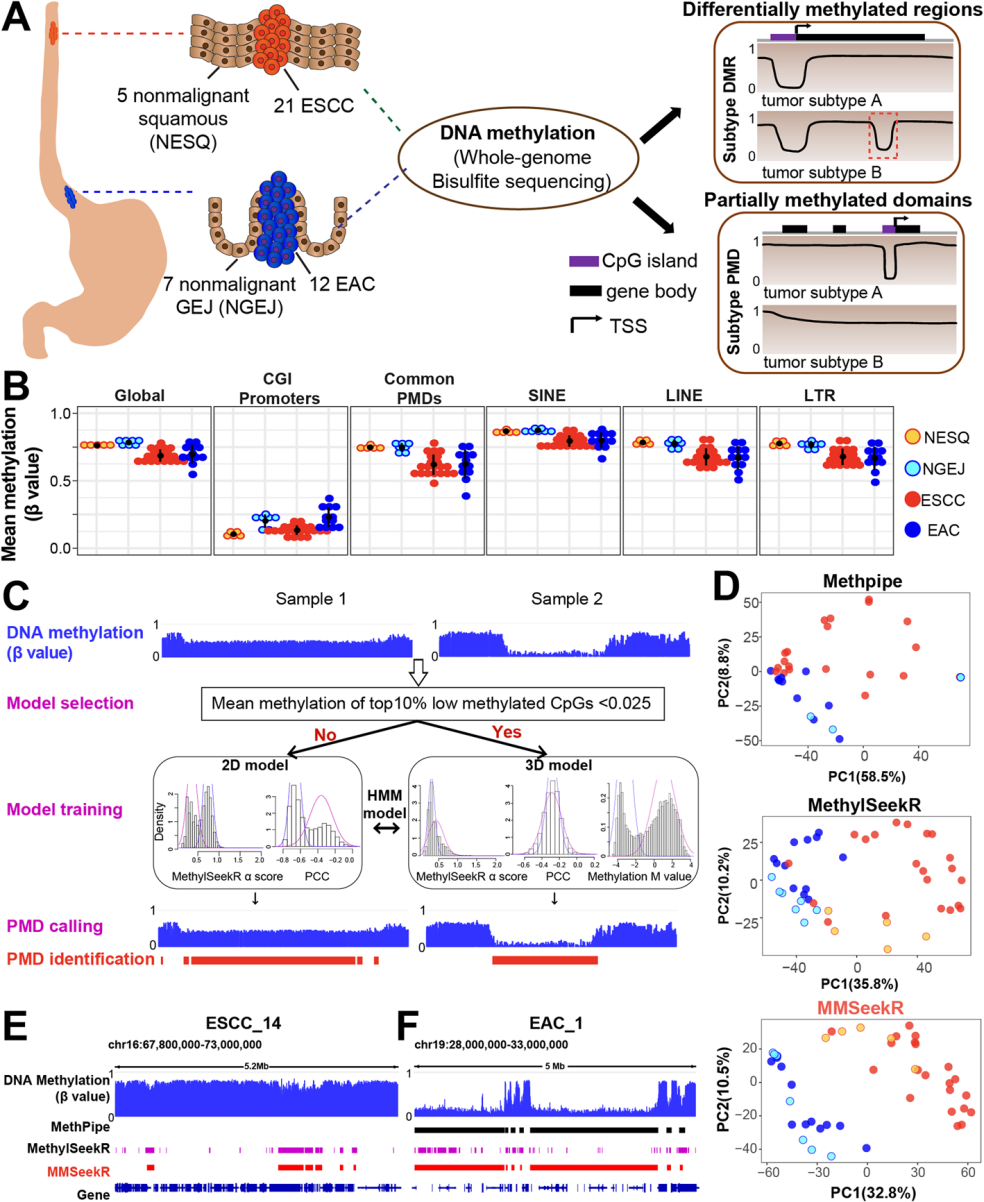
Identification of PMDs in esophageal samples by a sequence-aware multi-model PMD caller (MMSeekR). **A** A graphic model of the present study design. **B** Dot plots showing average methylation levels for all CpGs across the whole genome, CpGs within CGI promoters, common PMDs, SINE, LINE, and LTR in different samples. The annotations from Takai et al. [26] were used for CGI methylation quantification. **C** Development of a new PMD caller. The MethylSeekR *α* score measures the distribution of methylation levels in sliding windows with 201 consecutive CpGs across the genome. *α* score < 1 corresponds to a polarized distribution towards a high or low methylation level (that is, HMDs), while *α* score ≥ 1 corresponds to the distribution towards intermediate methylation levels (that is, PMDs). PCC shows the correlation between the predicted hypomethylation score based on a NN model, and the actual methylation level. A strong negative correlation indicates regions favoring PMDs, while weak/null correlation favors HMDs. **D** PCA analysis of 45 esophageal samples using the top 5000 most variable 30-kb tiles for the three PMD callers. **E**,** F** Representative windows showing PMDs successfully identified by MMSeekR but failed to be detected by either MethPipe (**E**) or MethylSeekR (**F**)

Several important questions on cell-type- and cancer-specific DMRs and PMDs await further characterization, including (i) the degree of the regional specificity of these domains (i.e., the proportions of DMR/PMD that are cell-type- and cancer-specific), (ii) the functional significance of DMRs and PMDs in cancer biology, and (iii) underlying mechanisms of the alteration of DMRs and PMDs during tumorigenesis. To address these questions, we performed analyses of WGBS data generated from a cohort of 45 esophageal samples, including 21 ESCC and 5 nonmalignant esophageal squamous (NESQ) tissues, as well as 12 EAC/GEJ tumors and 7 nonmalignant GEJ (NGEJ) tissues (Fig. 1A). We utilized NGEJ samples as the nonmalignant control for EAC/GEJ tumors considering recent studies suggesting an NGEJ/cardia origin of EAC based on genomics analyses of human samples [9, 25]. We chose esophageal cancer as the disease model considering that the two subtypes are developed from distinct cell-of-origins, and we hypothesized that characterization of their methylome profiles might reveal cell-type- and cancer-specific methylation changes, together with underlying epigenetic mechanisms.

## Results

### Development of a novel sequence-aware calling method to identify PMDs

To characterize the esophageal cancer methylome, we analyzed WGBS profiles of 45 esophageal samples from two different cancer subtypes and their corresponding nonmalignant tissues [27] (Fig. 1A, Additional file 1: Fig. S1A). All of the nonmalignant esophageal squamous (NESQ) tissues showed high inter-sample correlation despite that they were from two different cohorts (Additional file 1: Fig. S1B and Additional file 2: Table S1). To analyze the overall methylation pattern, we first investigated the methylation level at various genomic domains (Fig. 1B). As anticipated, both global hypomethylation (especially in common PMDs, defined as shared PMDs identified from 40 different cancer types [19]), and CGI promoter hypermethylation were observed in tumor samples. EAC tumors harbored notably higher methylation levels in CGI promoters than ESCC tumors, in line with TCGA results showing that gastrointestinal adenocarcinoma had higher frequency of CGI hypermethylation than cancers from most other tissues [28]. Interestingly, most NGEJ tissues showed higher CGI promoter methylation levels than NESQ tissues, and usually even higher than ESCC tumor samples. Similar to EAC, BE samples (a recognized precursor lesion of EAC) were reported to have a hypermethylation pattern at CGI promoters [7]. Since our NGEJ tissues were pathologically confirmed as inflammatory tissues but devoid of apparent BE, this result suggests that CGI hypermethylation may occur in inflamed GEJ. Interestingly, CGI hypermethylation has been observed in long-term-cultured colon organoids and cells upon prolonged exposure to cigarette smoke extract [29, 30]. These data suggest that prolonged extrinsic pressure may result in DNA methylation changes at CGIs. Repetitive elements, especially from the LINE and LTR classes, lost DNA methylation in tumors compared with nonmalignant tissues (Fig. 1B), which might be accompanied with the activation of repetitive elements in tumor samples [21, 31].

Considering the importance of PMDs in cancer biology [17, 19, 22, 23], we sought to characterize this epigenomic domain in depth. Computational tools have been developed for the identification of PMDs, including MethPipe [32] and MethylSeekR [33]. However, they sometimes fail or return unsatisfactory results for WGBS samples, either from tissues which have very slight hypomethylation (see Sample 1 in Fig. 1C) or tumors with near-complete methylation loss (see Sample 2 in Fig. 1C).

We recently used a deep learning neural network approach to establish universal sequence features that are almost entirely predictive of CpG methylation loss or retention in PMD regions of the human genome [34]. We hypothesized that utilizing sequence features associated with DNA methylation loss and exploiting the variation patterns among different CpGs within PMDs could improve the predictive models used in these tools (Additional file 1: Fig. S2A-D; see “Methods”). To this end, we developed a sequence-aware PMD calling method based on the Hidden Markov Model (HMM) used in MethylSeekR (Fig. 1C; see “Methods”), which was termed Multi-model PMD SeekR (MMSeekR). Importantly, using tumor samples from the Blueprint consortium, we showed that MMSeekR outperformed both MethylSeekR and MethPipe (Additional file 1: Fig. S2E-F). Indeed, MMSeekR successfully identified PMD fractions consistently across all samples and the Precision-Recall analysis showed that it had the highest F1 scores in almost all groups (using common PMDs as the reference for true positives, Additional file 1: Fig. S2F and Additional file 2: Table S2). While the score was sometimes only marginally better in MMSeekR, it was more consistent than MethylSeekR, which performed poorly on multiple cancer types (e.g., ALL, MM, AML), demonstrating that MMSeekR’s performance has high stability and consistency. PMD has been shown to exhibit cancer type specificity [22, 23], which can also be used to evaluate the performance of these methods. Notably, MMSeekR almost completely separated different cancer types, while both MethylSeekR and MethPipe produced much less clean separation (Additional file 1: Fig. S2G-H).

Encouraged by these results, we next applied MMSeekR to our esophageal samples (Additional file 1: Fig. S2I**-**J). Importantly, principal component analysis (PCA) using PMDs identified by three different methods again confirmed that MMSeekR outperformed MethylSeekR and MethPipe, completely separating EAC and ESCC samples (Fig. 1D, Additional file 1: Fig. S2K and Additional file 2: Table S3). Interestingly, nonmalignant samples clustered together with the corresponding cancer subtype. We also provided exemplary PMDs that failed to be identified by either MethPipe (Fig. 1E) or MethylSeekR (Fig. 1F).

### Characterization of shared and subtype-specific PMDs in esophageal samples

**Fig. 2:**
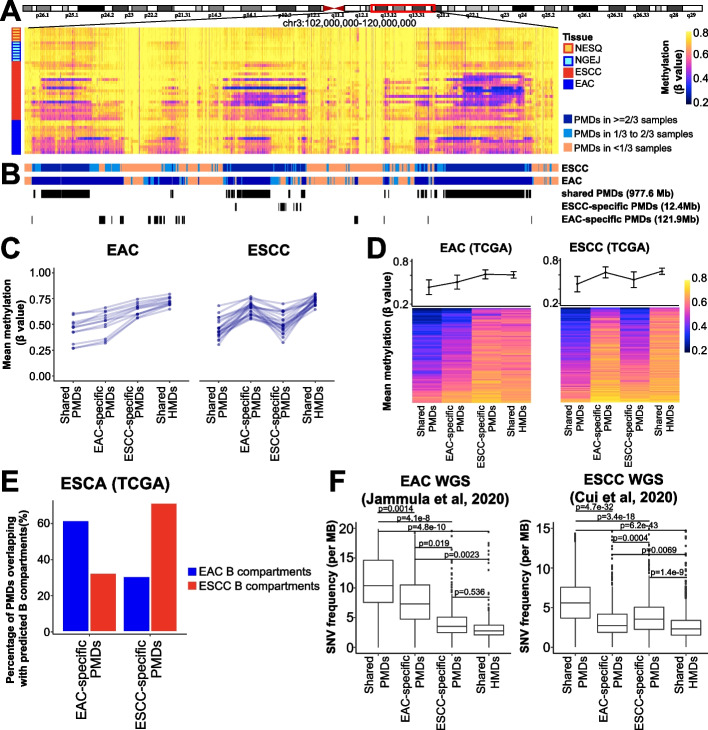
Characterization of shared and subtype-specific PMDs. **A** A representative window of DNA methylation profiles from 45 esophageal samples. Average methylation values are shown in consecutive and non-overlapping 10-kb tiles. CGI regions were masked using the annotation from Irizarry et al. [39]. **B** Different PMD categories were identified based on the frequency and overlap between the two esophageal cancer types. **C** Line plots showing average methylation levels for different PMD categories in esophageal tumors, where each line represents one sample. **D** Similar line plot patterns were observed using TCGA methylation datasets, showing the mean and standard deviation across samples. Each row in the heatmap below shows an individual sample. **E** Bar plots showing the percentage of WGBS PMDs overlapping with chromatin B compartments, which were predicted using TCGA methylation datasets and analyzed by minfi package. Methylation datasets in **D** and **E** are from the TCGA ESCA HM450k arrays, including 91 ESCC and 75 EAC samples. **F** Somatic mutation rates based on WGS in the indicated studies, calculated separately for each of the WGBS PMD categories. EAC WGS datasets: 276 samples; ESCC WGS datasets: 508 samples

We performed a genome-wide annotation of PMDs on a sample-by-sample basis (Fig. 2A). Consistent with our earlier report [19] and the genome-wide analysis (Fig. 1B), PMDs showed a slight decrease of DNA methylation in nonmalignant samples and lost methylation further in tumors. Notably, PMDs exhibited high inter-sample heterogeneity in both their depth (i.e., DNA methylation beta value) and breadth (i.e., genomic location). Indeed, the genome fraction covered by PMDs varied markedly across samples, ranging from 24.3 to 63.4% (Additional file 1: Fig. S3A). We categorized these methylation domains into 4 groups based on the frequencies of their occurrence in our cohort: shared PMDs, EAC-specific PMDs, ESCC-specific PMDs, and shared HMDs (Fig. 2B and Additional file 1: Fig. S3B**-**C; also see “Methods”). Interestingly, EAC-specific PMDs covered significantly more of the genome than ESCC-specific PMDs (121.9 Mb *vs.* 12.4 Mb). To verify our results, we used solo-WCGW CpGs, which lose methylation faster than other CpGs [19], to measure the average methylation loss within the 4 domain groups. In EAC samples, shared PMDs and EAC-specific PMDs had lower methylation levels than the other two groups, as expected (Fig. 2C, left panel). Reciprocally in ESCC samples, shared PMDs and ESCC-specific PMDs had lower methylation levels (Fig. 2C, right panel). Independent cohorts from either the TCGA (Fig. 2D) or other individual studies (Additional file 1: Fig. S3D-E) further validated these subtype-specific patterns of DNA methylation loss. Since PMDs are associated with the Hi-C B compartment [17, 23], we next mathematically modeled the A/B chromatin compartments for each esophageal cancer subtype using a method based on the HM450k array [35]. Indeed, subtype-specific PMDs were enriched in B compartments in a subtype-specific manner (Fig. 2E). By contrast, shared PMDs showed, as anticipated, no such specificity (Additional file 1: Fig. S3F). PMD regions were also reported to have higher somatic mutation rate compared with non-PMD regions in cancer [36, 37]. We analyzed the whole-genome sequencing (WGS) dataset from the OCCAMS (which has the largest number of EAC samples), finding a significantly higher somatic mutation rate in EAC-specific PMDs than in either ESCC-specific PMDs or HMDs (Fig. 2F, left panel). A reciprocal pattern was observed in the largest ESCC WGS cohort (Fig. 2F, right panel). We further investigated the mutational signatures using the method MutationalPatterns [38]. While most of the mutational signatures had comparable weight between genome-wide mutations and PMD-restricted mutations, we noted a consistent and conspicuous decrease of SBS40 and SBS5 signatures (both are associated with age) in cancer-specific PMDs in both ESCC and EAC tumors (Additional file 1: Fig. S3G). This result is interesting and supports our findings on PMDs: since cancer-specific PMDs occur during tumor development, which is independent of age. Therefore, mutations within cancer-specific PMDs display much lower age-related signatures than genome-wide mutations.

We also correlated the methylation levels of subtype-specific PMDs to each of risk factors and clinicopathological parameters using HM450k datasets from the TCGA ESCA project. None of these factors, including age, smoking history, alcohol consumption, lymph node metastasis, and clinical stage, had significant impact on subtype-PMDs (Additional file 1: Fig. S3H). Another independent WGBS dataset (PRJNA523898, *n* = 42) again confirmed that there was no association between ESCC-specific PMDs with either age, clinical stage, or lymph node metastasis (Additional file 1: Fig. S3I).

**Fig. 3:**
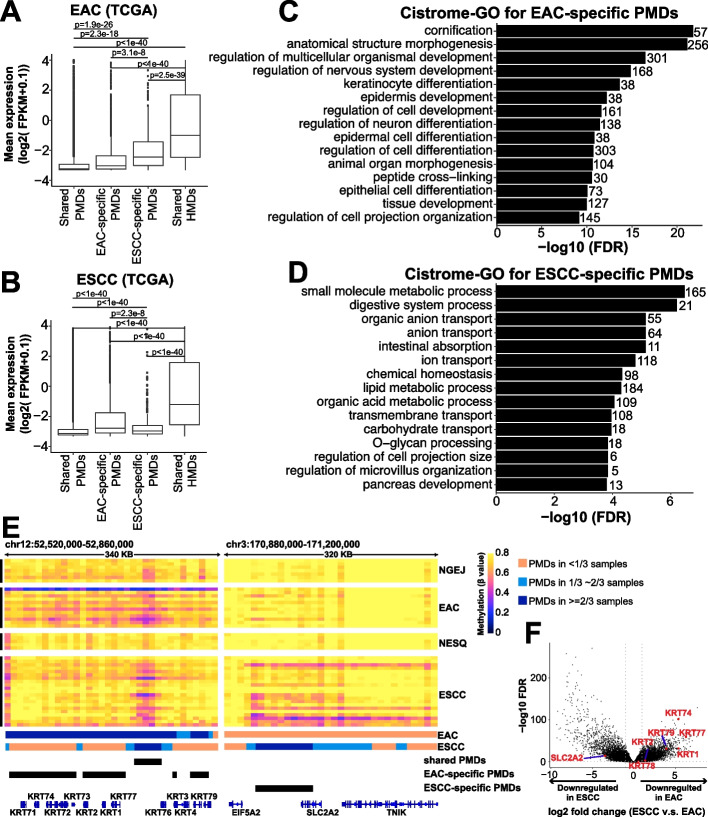
Subtype-specific PMDs control cell-type-specific functions. **A**,** B** In both EAC (**A**) and ESCC (**B**), genes covered by PMDs are expressed at lower levels than those in non-PMDs in a cancer-specific manner. **C**, **D** Cistrome-GO enrichment analyses using either EAC-specific (**C**) or ESCC-specific (**D**) PMDs and the downregulated genes within them. The top 15 most significant pathways are shown, and the number of genes enriched in each pathway is shown on the right. **E** Two representative genome windows showing the methylation profiles of EAC-specific (left panel) and ESCC-specific PMDs (right panel). CGI regions were masked using the annotation from Irizarry et al. [39]. **F** Volcano plots showing that genes residing within genome domains in **E** are downregulated in corresponding cancer subtypes. The differentially expressed genes were identified with the average expression level (FPKM) ≥ 0.1, adjusted *p*-value < 0.05 and absolute fold-change > 2. Expression RNA-seq in **C**, **D**, **F** are from the TCGA ESCA project, including 76 ESCC and 78 EAC samples

At the transcription level, PMDs are reported to be less transcriptionally active than HMDs. We confirmed that subtype-specific PMDs were associated with low levels of gene expression specifically in the corresponding subtypes (Fig. 3A, B). To explore the biological implication of subtype-specific PMDs, we performed Cistrome-GO analysis using genes which were under-expressed in the subtype-specific PMD regions, finding that biological processes characteristic for the other subtype were enriched and repressed (Fig. 3C, D). Specifically, pathways of cornification, keratinocyte differentiation, and epidermis development, which are central to squamous cell differentiation and function, were enriched and inactive in EAC-specific PMDs (Fig. 3C). For example, many keratinocyte-specific genes were clustered within EAC-specific PMDs (Fig. 3E, left panel) and downregulated in EAC tumors (Fig. 3F). On the other hand, pathways important for gastrointestinal cell function, such as digestive system process, intestinal absorption, lipid metabolic process, and O − glycan processing, were enriched and suppressed in ESCC-specific PMDs (Fig. 3D). The right panel of Fig. 3E shows as an example that SLC2A2, which contributes to digestive system process and absorption, was located in ESCC-specific PMDs and downregulated in ESCC samples (Fig. 3F). These results suggest that subtype-specific PMDs contain inactive genes which are associated with cell-type-specific functions.

### H3K36me2 is inversely associated with PMDs in a cell-type-specific manner

**Fig. 4:**
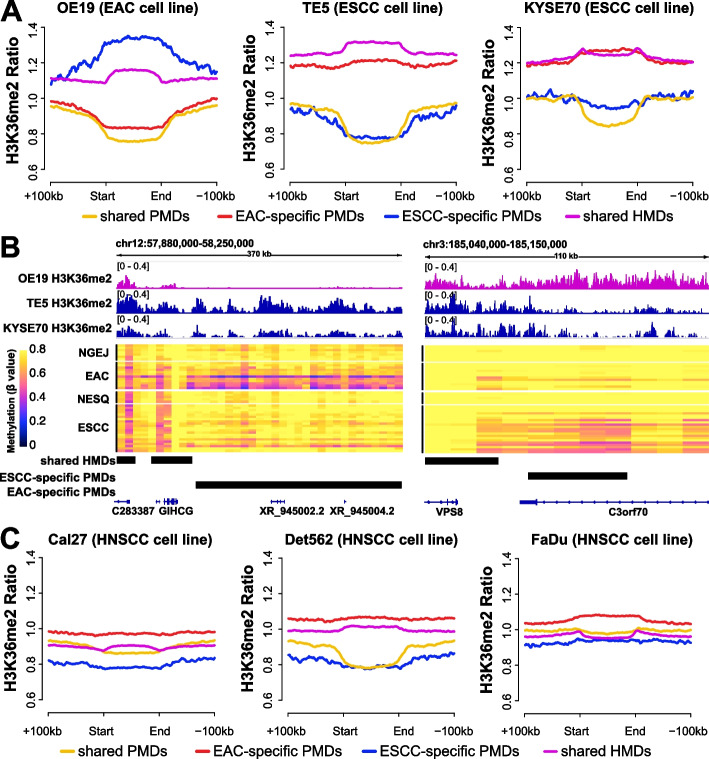
The H3K36me2 mark is inversely associated with PMDs in a cell-type-specific manner.** A** Aggregation plots of H3K36me2 ChIP-seq levels in esophageal cancer cell lines across four different PMD categories: shared PMDs, EAC-specific PMDs, ESCC-specific PMDs, shared HMDs. **B** Representative genomic loci showing H3K36me2 signal from ChIP-seq, and subtype-specific PMDs from WGBS data. CGI regions were masked using the annotation from Irizarry et al. [39]. **C** Aggregation plots of H3K36me2 ChIP-seq levels in HNSCC cell lines across four different PMD categories. H3K36me2 ChIP-seq datasets were obtained from GSE149670. H3K36me2 signals in **A**, **C** were normalized by the CPM ratio of IP over Input in 5-kb windows

Both H3K36me2 and H3K36me3 were observed to recruit DNA methyltransferases (DNMT3A [40] and DNMT3B [41], respectively) to maintain DNA methylation levels in large chromatin domains. H3K36me3 is enriched in gene bodies of active transcripts, while H3K36me2 covers larger multi-gene domains. Indeed, we have previously shown that the deposition of H3K36me3 is inversely associated with PMD distribution [19]. Here, we further hypothesized that H3K36me2 also contributed to maintaining DNA methylation levels, and the histone modification by this mark might affect the genomic distribution of PMDs and HMDs. To test this, we performed H3K36me2 ChIP-seq in both EAC and ESCC cell lines. Indeed, shared HMDs (purple line) showed high H3K36me2 intensity in both cell types, while shared PMDs (yellow line) exhibited the lowest signals (Fig. 4A). EAC-specific PMDs (red line) had low H3K36me2 levels in EAC cells but high H3K36me2 levels in ESCC cells. The reciprocal pattern was observed in ESCC-specific PMDs (blue line). For example, H3K36me2 signals were undetectable in an EAC-specific PMD covering the loci of *XR_945002.2* and *XR_945004.2* in EAC cells, but were strong in ESCC (Fig. 4B, right panel). On the other hand, shared HMDs such as the one covering the *VSP8* gene were decorated highly with H3K36me2 in both cell types (Fig. 4B, left panel).

To further verify these results, we interrogated public H3K36me2 ChIP-seq data from HNSCC cell lines (squamous cancer highly similar to ESCC in terms of cell-of-origin and epigenome). Indeed, a similar pattern of H3K36me2 distribution to ESCC was observed in Cal27 and Det562 HNSCC cells. Specifically, both shared PMDs and ESCC-specific PMDs harbored low signals in HNSCC cell lines, while high H3K36me2 levels were found in HMDs and EAC-specific PMDs (Fig. 4C). However, FaDu appeared to be an outlier, showing invariably high levels across different regions (Fig. 4C), which warrants further investigation. Together, these results demonstrate a prominent depletion of H3K36me2 mark in PMDs in a cell-type-specific manner, which is likely owing to the finding that H3K36me2 promotes the maintenance of DNA methylation by recruiting DNMT3A.

### Subtype-specific differentially methylated regions (DMRs) in esophageal cancer

We next sought to investigate differentially methylated regions (DMRs) at small genomic scales, given their direct roles in transcriptional regulation. However, our above results suggest an overwhelming, global effect of PMD hypomethylation in tumor samples, which can strongly affect the calling of focal DMRs. Indeed, PCA analysis of the most variable CpGs genome-wide revealed that PC1, the most significant component, was clearly driven by methylation loss at PMDs (Additional file 1: Fig. S4A).

To factor out the effect of PMD hypomethylation, we masked any PMD found within two-thirds of either EAC or ESCC samples (Additional file 1: Fig. S4B). We re-performed the PCA analysis, finding that the two cancer subtypes were completely separated by PC1, which was the most significant component and accounted for 42.2% of the total methylation variance (Additional file 1: Fig. S4C, left panel). In addition, nonmalignant and tumor samples were separated along PC2, and all NESQ samples were clustered closely together despite being generated from two different cohorts. Notably, this approach removed most correlation with the global methylation level (Additional file 1: Fig. S4C, right panel). Thus, it is critical to remove the effects of global hypomethylation when investigating cancer-associated methylation features outside PMDs.

**Fig. 5:**
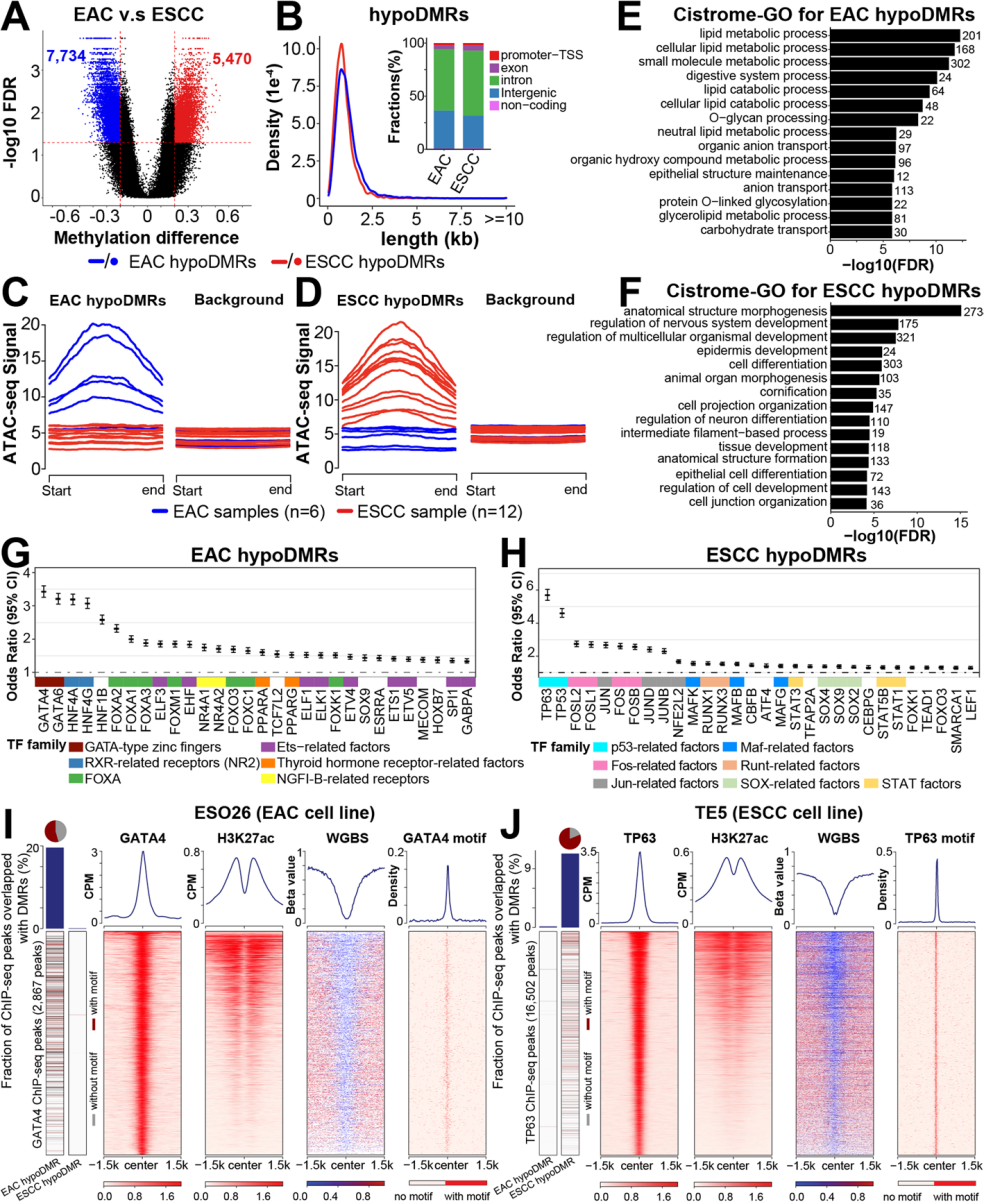
Subtype-specific DMRs in esophageal cancer.** A** Cancer hypoDMRs were identified from the comparison between EAC and ESCC tumors. Regions with FDR < 0.05 and absolute delta methylation levels > 0.2 were identified as DMRs. **B** Density plots showing the size distribution of hypoDMRs; stacked bar plots displaying fractions of hypoDMRs that overlap with different genomic features. **C**, **D** Aggregation plots of ATAC-seq signals from esophageal cancer samples within EAC (**C**) or ESCC (**D**) hypoDMRs or random genomic regions (background), which contained 10 times randomly selected regions with the same CpG density. ATAC-seq signals were obtained from the TCGA and normalized with the CPM method. **E**, **F** Cistrome-GO enrichment analyses using EAC (**E**) or ESCC (**F**) hypoDMRs and upregulated genes in the corresponding subtype. Top 15 most significant pathways are shown. The number of genes enriched in each pathway is shown on the right. Expression datasets are from the TCGA ESCA project. **G**, **H** Transcription-factor-binding motif sequences were identified by the ELMER [47] method using EAC (**G**) or ESCC (**H**) hypoDMRs as the foreground and random regions as the background. The annotation of the TF family is from the TFClass database [48]. **I**, **J** The most strongly enriched TFs in EAC (GATA4) (**I**) and ESCC (TP63) (**J**) were chosen for the experimental validation, using TF ChIP-seq, H3K27ac ChIP-seq, and WGBS in matched cell lines. Peaks overlapping with subtype hypoDMRs are shown on the left; the percentages of overlapped peaks are expressed in the column plots. The pie charts at the upper left corner denote the proportion of peaks with TF binding motifs over all peaks overlapping with subtype hypoDMRs. Cell line ChIP-seq and WGBS datasets are listed in the “ Methods” section

We next identified DMRs between EAC and ESCC samples within the PMD-subtracted genome described above (~ 46.5% of the genome). Under the cutoff of *q* value < 0.05 and absolute delta methylation change > 0.2, a total of 7734 DMRs were hypomethylated in EAC and 5470 in ESCC (Fig. 5A). As expected, hypomethylated DMRs (hypoDMRs) had low average methylation levels in corresponding subtypes (Additional file 1: Fig. S4D-E). The majority of DMRs were about 1–2 kb long and located mostly in intronic and intergenic regions (Fig. 5B), similar to that of the random background (Additional file 1: Fig. S4F). To investigate the epigenomic characteristics of hypoDMRs, we systematically evaluated the chromatin accessibility at these regions, using the ATAC-seq data from the TCGA [42] and H3K27ac ChIP-seq data from previous studies [43–46]. Relative to random background regions, EAC hypoDMRs were accessible exclusively in EAC samples, and ESCC hypoDMRs exclusively in ESCC samples (Fig. 5C,D). Additionally, EAC hypoDMRs had high H3K27ac signals in 70% (5/7) of EAC cell lines (Additional file 1: Fig. S4G). A similar observation was made in ESCC cell lines (Additional file 1: Fig. S4H). These data demonstrate that hypoDMR regions are associated with accessible chromatin and active histone marks. Similar with subtype-specific PMDs, none of clinicopathological parameters (including age, smoking history, alcohol consumption, lymph node metastasis, and clinical stage) showed influence on subtype-specific DMRs (Additional file 1: Fig. S4I-J).

To explore the relevance of DMRs in gene transcription, we assigned each hypoDMR to the closest genes annotated by HOMER [49, 50], and performed correlational analyses using TCGA transcriptomic data of esophageal cancers. Consistent with prior findings [49], about 30% (3986/13,204) of the DMRs were associated with differentially expressed genes (Additional file 1: Fig. S4K). Expectedly, an inverse correlation between DNA methylation and gene expression accounted for the majority (~ 59%) of these associations, and these DMRs had a larger overlap with promoter and enhancer regions (Additional file 1: Fig. S4L). Importantly, functional annotation using the Cistrome-GO method revealed that subtype hypoDMRs were enriched in cell-type-specific biological processes. For example, lipid metabolic process, digestive system process, and O − glycan processing, which are housekeeping functions for gastrointestinal columnar cells, were specifically enriched in EAC hypoDMRs (Fig. 5E). On the other hand, epidermis development, cornification, and epithelial cell differentiation, which are unique to squamous cells, were enriched in ESCC hypoDMRs (Fig. 5F). These results indicate that a large number of hypoDMRs regulate the transcription of cell-type-specific genes.

We next performed sequence motif enrichment analysis of hypoDMRs, which have previously been associated with transcription-factor-binding sites [17, 22, 51]. A number of known esophageal cell-specific transcription factors were identified, including GATA4/6, HNF4A/G, HNF1B, ELF3, EHF in EAC [43, 52, 53], and TP63, SOX2, and MAFB in ESCC [45, 54] (Fig. 5G,H). To validate these results, we focused on the top-ranking transcription factors (GATA4 for EAC, TP63 for ESCC). Specifically, we performed WGBS in an EAC cell line (ESO26) where we previously generated ChIP-seq data for GATA4 and H3K27ac. Indeed, GATA4 ChIP-seq peaks were associated with high H3K27ac signal, DNA hypomethylation, and GATA4 binding motif sequence (Fig. 5I). Moreover, ~ 20% of GATA4 peaks overlapped with EAC hypoDMRs. Additionally, 54.5% of these hypoDMRs contained GATA4 motif sequences (pie chart, upper left corner). In sharp contrast, almost no GATA4 peaks were found in ESCC hypoDMRs (Fig. 5I, left bars). We similarly performed WGBS on an ESCC cell line (TE5) and analyzed TP63 ChIP-Seq data that we generated in the same sample. We noted consistent patterns and significant overlap with ESCC hypoDMRs in this ESCC-specific transcription factor, and almost no overlap with EAC hypoDMRs (Fig. 5J). These results demonstrate that subtype-specific DMRs are occupied by cell-type-specific transcription factors and contribute to regulation of cell-type-specific functions.

### Identification of tumor-specific hypoDMRs

**Fig. 6:**
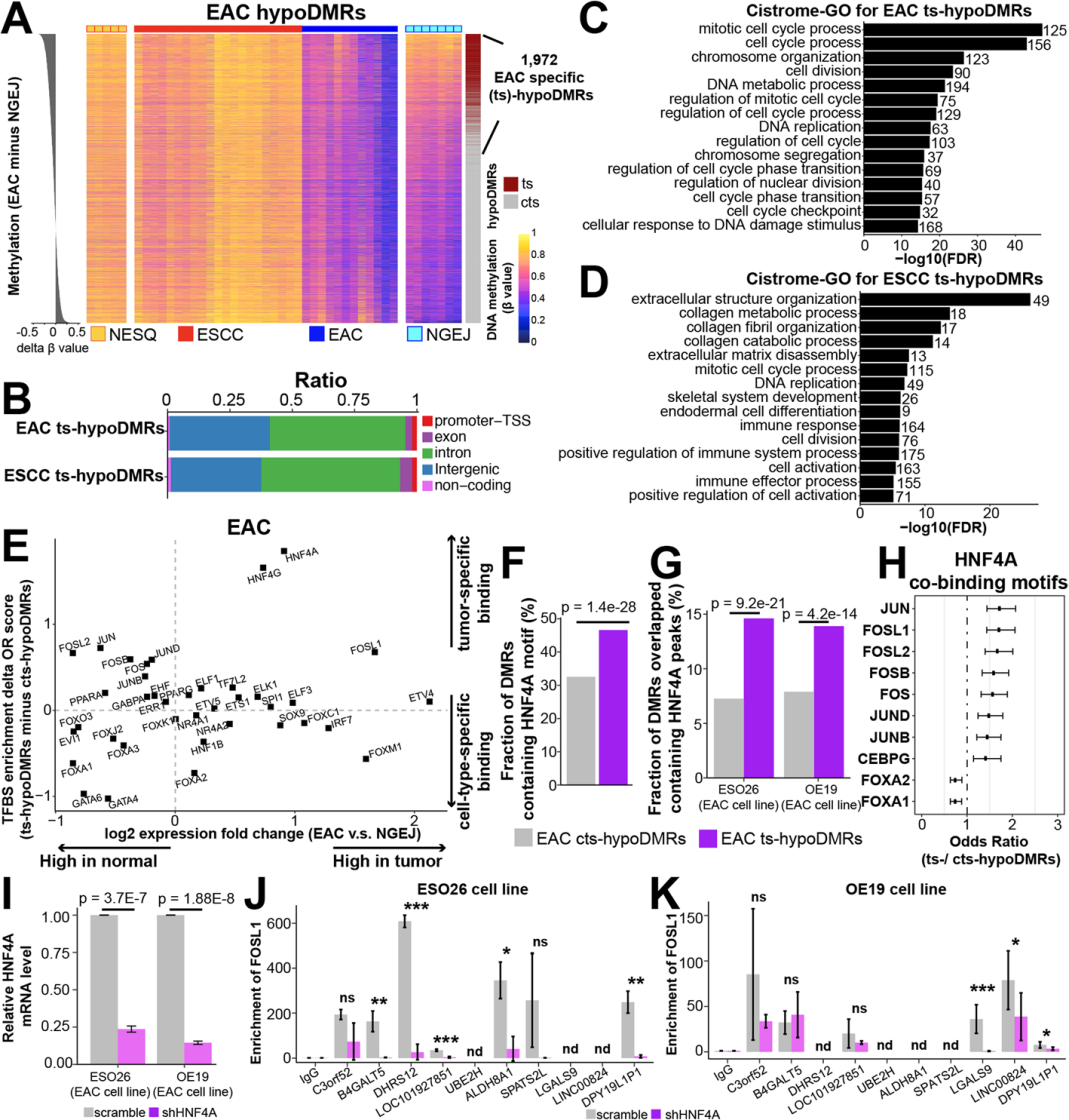
Identification of tumor-specific hypoDMRs.** A** Heatmaps showing DNA methylation levels for each EAC hypoDMR. Each column denotes one sample and the row was ordered by the delta mean methylation between EAC and NGEJ (left). EAC ts-hypoDMRs were identified using a one-tailed *t* test between EAC tumor and NGEJ samples (right) with the FDR cutoff < 0.05. **B** Stacked bar plots showing fractions of ts-hypoDMRs that overlap with different genomic features. **C**, **D** Cistrome-GO enrichment analyses using either EAC (**C**) or ESCC (**D**) ts-hypoDMRs and the upregulated genes in each subtype compared with corresponding nonmalignant samples. Top 15 most significant pathways are shown. The transcriptomic data of esophageal cancer from the TCGA consortium and GSE149609. **E** Scatter plots showing transcription-factor-binding sites that were enriched in EAC ts-hypoDMRs compared with cts-hypoDMRs. The *X* axis represents the expression fold change between EAC and matched nonmalignant GEJ samples. The *Y* axis shows the delta enrichment score of transcription-factor-binding sites between EAC ts- and cts-hypoDMRs. Expression data were from the TCGA and motif enrichment analyses were performed by the ELMER method. **F** EAC ts-hypoDMRs contained significantly more HNF4A-recognition motifs compared with cts-hypoDMRs. **G** More HNF4A peaks overlapped with ts-hypoDMRs than cts-hypoDMRs. Peaks were called from HNF4A ChIP-seq in ESO26 (GSE132813) and OE19 cell lines (E-MTAB-6858). **H** HNF4A was predicted to co-occupy with the AP-1 family in ts-hypoDMRs, while with FOXA1/2 in cts-hypoDMRs. Sequence motif analysis was performed using ts- *vs.* cts-hypoDMRs containing HNF4A motifs. Significant transcription factors with FDR < 0.05 are shown. OR value over 1 represents higher enrichment in ts-hypoDMRs, while below 1 represents higher enrichment in cts-hypoDMRs. **I** qPCR experiments measuring HNF4A mRNA expression in the scramble shRNA vs. shHNF4A group in ESO26 and OE19 cell lines. **J**, **K** FOSL1 ChIP-qPCR assays were performed in ESO26 (**J**) and OE19 (**K**) cells, in either the scramble shRNA or shHNF4A group. IgG was used as a negative control antibody. The number of biological replicates is 3. *p*-values were determined by a two-sided *t* test. ***p < 0.001; **p < 0.01; *p < 0.05; ns, not significant; nd, not detectable

To identify tumor-specific hypoDMRs from the above subtype-specific DMRs and to investigate their role in cancer biology, we next performed a methylation comparison between tumors and their corresponding nonmalignant samples for each hypoDMR. We found that 25.5% (1972/7734) of EAC hypoDMRs (Fig. 6A) and 12.0% (654/5470) of ESCC hypoDMRs (Additional file 1: Fig. S5A) had significantly lower (FDR < 0.05) methylation levels in tumors than corresponding nonmalignant samples, which were referred to as “tumor-specific hypoDMRs (ts-hypoDMRs)”, while the rest were referred to as “cell-type-specific DMRs (cts-hypoDMRs)”. Ts-hypoDRMs were distributed in both intergenic and intronic domains, similar to hypoDMRs overall and the random background (Fig. 6B and Additional file 1: Fig. S5B). Between 18.0 and 21.4% of ts-hypoDMRs were correlated with the expression of nearest genes (Additional file 1: Fig. S5C**-**D). Importantly, ts-hypoDMRs were strongly enriched in cancer-related pathways such as cell cycle progression (in both EAC and ESCC), and extracellular structure organization in ESCC (Fig. 6C**-**D). These data suggest that ts-hypoDMRs are associated with genes which contribute to tumor-specific functions.

The identification of ts-hypoDMRs and cts-hypoDMRs allowed us to further investigate properties of tumor-specific regulatory regions *vs.* cell-type-specific regulatory regions. This is particularly helpful for the epigenetic understanding of ESCC and EAC, which contain both tumor- and cell-type-specific features. In addition, lineage-specific developmental factors have been shown to promote malignant cell states [55, 56], and thus it is important to distinguish their functional contribution to normal development *vs.* cancer biology. To this end, we performed motif enrichment analysis to identify transcription-factor-binding sites that were unique to either ts- or cts-hypoDMRs, and integrated expression patterns of the corresponding transcription factors. For EAC, this approach revealed cancer-upregulated transcription factors which favored binding ts-hypoDMRs, including HNF4A, HNF4G, and FOSL1 (upper right corner of Fig. 6E). In comparison, the lower left corner of Fig. 6E contained cancer-downregulated transcription factors which preferred occupying cts-hypoDMRs, including GATA4/6 and FOXA, which are well-recognized for their key roles in the development of gastrointestinal cell lineage [57, 58]. The top factor for ts-hypoDMR, HNF4A, had its binding motif in 46.6% ts-hypoDMRs but only 32.6% cts-hypoDMRs (Fig. 6F). Indeed, ChIP-seq data of HNF4A in EAC cell lines (ESO26 and OE19) validated this bias: HNF4A binding peaks overlapped with 14.2% ts-hypoDMRs but only 7.6% cts-hypoDMRs (Fig. 6G). To identify factors that may cooperatively bind with HNF4A specifically to hypoDMRs, we performed enrichment analyses restricted within HNF4A-motif-containing hypoDMRs. Interestingly, AP-1 motifs (such as JUN, FOSL1, FOSL2, and FOSB) were enriched in these HNF4A^+^ ts-hypoDMRs, while FOXA1/2 in cts-hypoDMRs (Fig. 6H). This distinct pattern of co-occurring motifs between ts- and cts-hypoDMRs in EAC is noteworthy, considering that AP-1 family transcription factors contribute to EAC tumor development [59] while FOXA1/2 are required for normal gastrointestinal cell development [58].

FOSL1 was particularly interesting, because it was the only highly overexpressed AP-1 factor in EAC tumors vs. nonmalignant samples (Fig. 6E). To validate the involvement of FOSL1 experimentally, we randomly chose 10 HNF4A-occupying ts-hypoDMRs which also contained the FOSL1 motif sequence for ChIP-qPCR assay. The majority of these regions were indeed occupied by FOSL1 in ESO26 and OE19 EAC cells, and importantly, the binding of FOSL1 was significantly reduced in a subset of occupied regions upon the knockdown of HNF4A (Fig. 6I–K). This result suggests that HNF4A is functionally required for the occupancy of FOSL1 on a subset of EAC ts-hypoDMRs, a possible mechanism underlying the strong co-occurrence of their motif sequences in these epigenetic regions.

A parallel analysis was performed in ESCC, which identified a number of tumor-specific factors, including RUNX1/3, SOX2/4, and CEBPA/B (Additional file 1: Fig. S5E). In addition, we performed similar analyses to identify ts-hyperDMRs (Additional file 1: Fig. S6A**-**C), which showed no enrichment in cancer-related pathways (Additional file 1: Fig. S6D**-**E).

### PMDs and hypoDMRs exhibit strong cell-type-specific epigenomic features

**Fig. 7:**
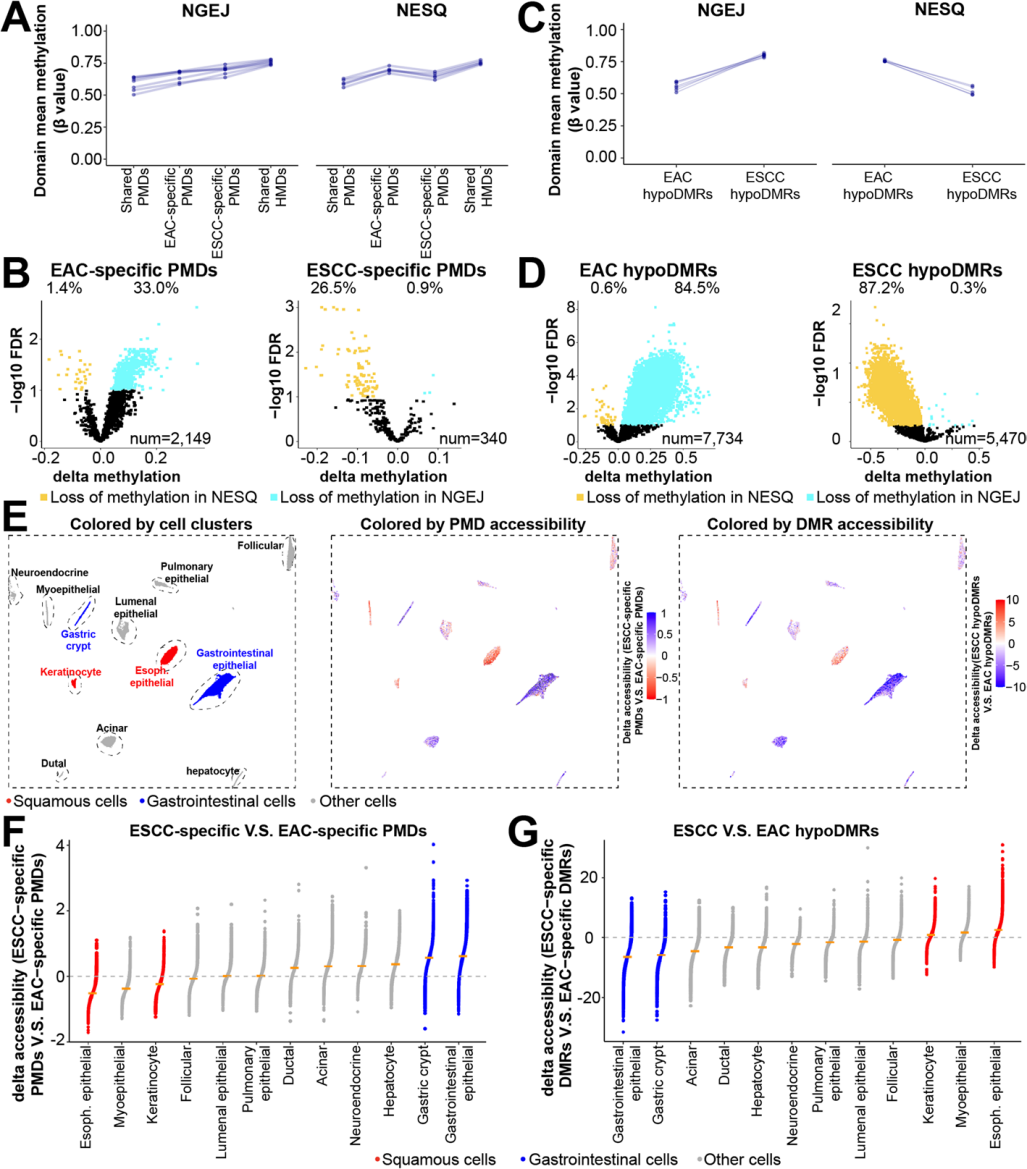
PMDs and hypoDMRs exhibit strong cell-type-specific epigenomic features.** A** Line plots showing average methylation levels for different PMD or **C** hypoDMR categories comparing two types of nonmalignant esophageal samples; these changes in nonmalignant samples are similar to those seen in tumors (Fig. 2C, Additional file 1: Fig. S4D-E). **B** Volcano plots showing average methylation levels for different PMD or **D** hypoDMR categories in nonmalignant esophageal samples. Regions with significant differences were determined by two-tailed *t* test with the FDR cutoff < 0.1. **E** UMAP plots showing cell clusters (left), ATAC-seq levels in ESCC- *vs.* EAC-specific PMDs (middle) or in ESCC- *vs.* EAC-specific hypoDMRs (right). Single-cell ATAC-seq values and the cluster scheme were from Zhang et al. Total cell number is 146,305. **F**, **G** Dot plots showing, at the sample level, delta ATAC-seq values in ESCC- *vs.* EAC-specific PMDs (**F**) or in ESCC- *vs.* EAC-specific hypoDMRs (**G**)

The above data identified both cell-type- and cancer-specific methylation differences in tumor hypoDMRs, and we next asked whether tumor PMDs likewise harbor both of these two types of methylation differences. In subtype-specific PMDs that were defined based on tumor methylomes alone, nonmalignant tissues notably exhibited the same pattern of methylation changes as their malignant counterparts (Fig. 7A). For example, EAC-specific PMDs had low methylation levels in NGEJ but high in NESQ (Fig. 7A, left), and a reciprocal pattern was found in ESCC-specific PMDs (Fig. 7A, right). Statistically, a large subset of subtype-specific PMDs (33.0% for EAC and 26.5% for ESCC) were already hypomethylated in their respective nonmalignant samples (Fig. 7B). The same analyses for hypoDMRs confirmed that more than 80% of subtype hypoDMRs significantly decreased DNA methylation in their corresponding nonmalignant samples (Fig. 7C,D). These data demonstrate that a substantial fraction of both subtype-specific PMDs and hypoDMRs identified from tumor samples reflect methylation differences present in normal counterparts. Nonetheless, while the genomic locations of PMDs are established in normal samples, the degree of methylation loss is significantly higher in tumors (Fig. 2C and Additional file 1: Fig. S4D-E).

To understand further PMDs and hypoDMRs in normal samples, we analyzed public single-cell ATAC-seq data from 146,305 normal epithelial cells across 24 tissues (including esophageal samples) [60], by measuring the chromatin accessibility of our subtype-specific PMDs or hypoDMRs. This is premised on the fact that focal ATAC-seq peaks are almost always DNA demethylated [42], and reduced ATAC-seq signals measured in large genomic windows reflect the Hi-C B compartment which results in PMD hypomethylation [17, 23]. The published single-cell unsupervised clustering contains a cluster of esophageal squamous epithelial cells (red dots in Fig. 7E, left panel), the recognized cell-of-origin for ESCC. With respect to EAC, although its cell-of-origin is still under intense investigation, the epigenome is likely close to gastrointestinal epithelial cells (blue dots Fig. 7E, left panel). Importantly, normal esophageal squamous cells showed the lowest chromatin accessibility in ESCC-specific PMDs; reciprocally, normal gastrointestinal epithelial cells had the lowest ATAC-Seq signals in EAC-specific PMDs (Fig. 7E, middle panel; quantified in Fig. 7F). In addition, keratinocytes, which belong to squamous cell type, also had low ATAC-Seq signals in ESCC-specific PMDs. In sharp contrast to subtype-specific PMDs, no difference was observed in either shared PMDs or HMDs in this single-cell analysis (Additional file 1: Fig. S7A). We performed the same analysis for hypoDMRs, finding that ESCC hypoDMRs had the highest accessibility in squamous cells while EAC hypoDMRs were more open in gastrointestinal epithelial cells (Fig. 7E, right panel; quantified in Fig. 7G). These single-cell results confirmed that both PMDs and hypoDMRs have strong normal cell-type specificity.

### Pan-cancer analysis of subtype-specific PMDs and hypoDMRs

**Fig. 8:**
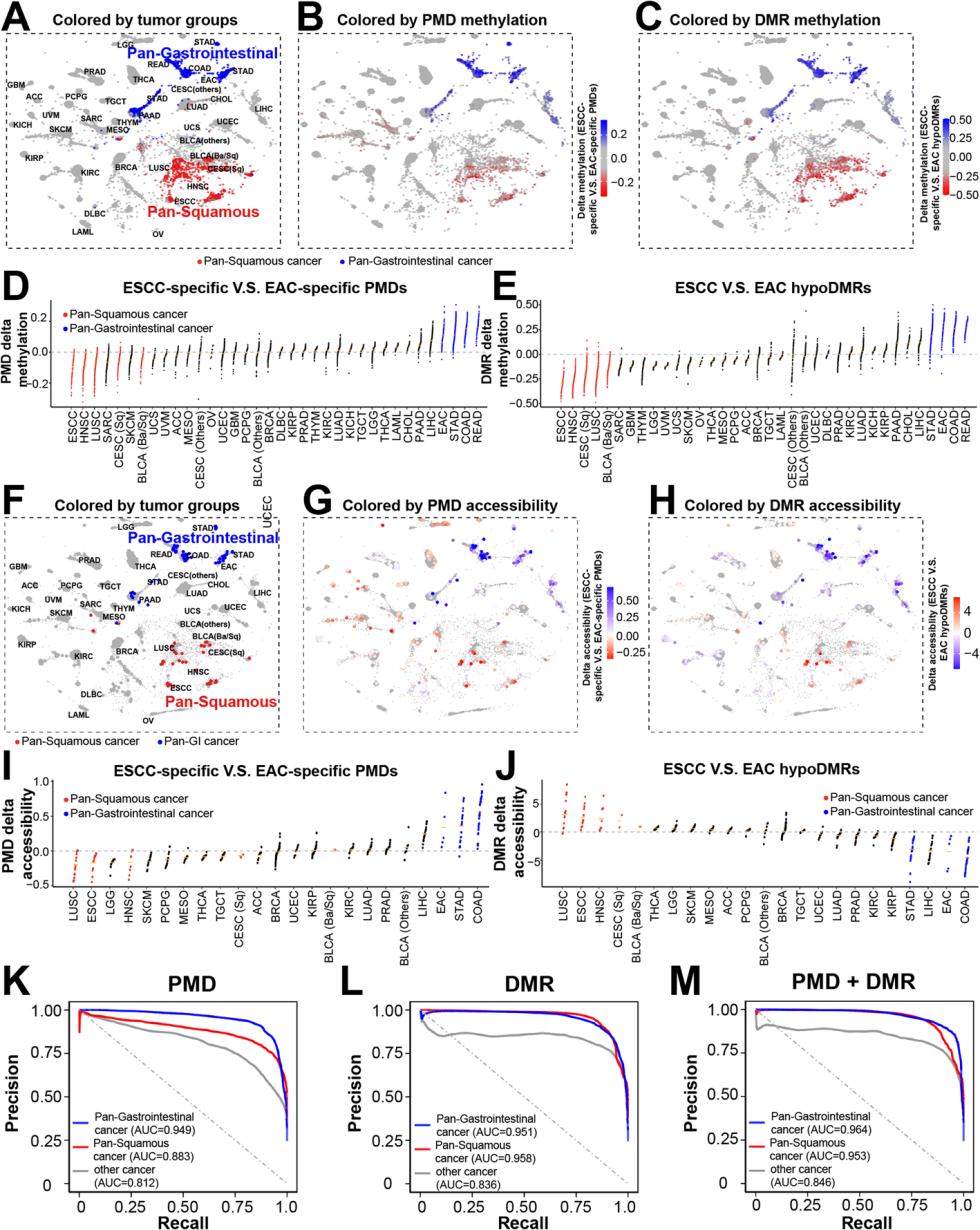
Analyses of PMDs and hypoDMRs in pan-cancer datasets. **A**–**C** TCGA tumormap showing cancer type clusters (**A**), DNA methylation levels in ESCC- *vs.* EAC-specific PMDs (**B**), or in ESCC- *vs.* EAC-specific hypoDMRs (**C**). DNA methylation data were obtained from the TCGA project. The TCGA-based clustering scheme denotes pan-gastrointestinal cancers (COAD, READ, STAD, and EAC) and pan-squamous cancers (ESCC, HNSC, LUSC and a subset of CESC and BLCA) are shown **A**. The number of samples is 8915. The detailed study name of TCGA study abbreviations are listed in https://gdc.cancer.gov/resources-tcga-users/tcga-code-tables/tcga-study-abbreviations. **D**, **E** Dot plot quantification of the methylation differences in **B** and **C**, respectively. **F** t-SNE plots showing cancer type clusters, **G** ATAC-seq levels in ESCC- *vs.* EAC-specific PMDs or in **H** ESCC- *vs.* EAC-specific hypoDMRs across tumor samples. ATAC-seq data were downloaded from the TCGA project. The number of samples is 362. **I**, **J** Dot plots quantification of the ATAC-seq values in **G** and **H**, respectively. **K**–**M** Precision-recall curves for three-class classification characterized by the average methylation of subtype-specific PMDs (**K**), DMRs (**L**), or both (**M**) respectively. The training datasets were from panels **B** and **C**

The above results also suggest that PMDs and hypoDMRs that we identified in ESCC and EAC may be shared with other squamous and gastrointestinal adenocarcinomas, respectively. To test this, we analyzed TCGA pan-cancer samples, since the TCGA multi-omic clustering scheme [61] has identified the pan-gastrointestinal cluster (adenocarcinomas from esophagus, stomach, and colon, blue samples in Fig. 8A) and the pan-squamous cluster (squamous cancers from esophagus, head and neck, lung, cervix, and bladder, red samples in Fig. 8A). We first measured the methylation changes between subtype-specific PMDs and hypoDMRs across all 33 cancer types (Fig. 8B–E). Importantly, most pan-gastrointestinal tumors lost DNA methylation in EAC-specific PMDs, while most pan-squamous tumors had reduced methylation in ESCC-specific PMDs (Fig. 8B**-**D). Highly consistent results were observed in subtype hypoDMRs (Fig. 8C, E). In contrast, no specific pattern was found in shared PMDs and HMDs (Additional file 1: Fig. S7B), as anticipated.

We next analyzed the ATAC-seq data, which is available from a small subset of TCGA bulk tumors [42], shown based on multi-omic clustering from ref [61] in Fig. 8F. Importantly, consistent with the single-cell ATAC-Seq results from healthy tissues, pan-squamous cancers showed the lowest chromatin accessibility in ESCC-specific PMDs and highest accessibility in ESCC hypoDMRs, and the reciprocal results were obtained in pan-gastrointestinal cancers (Fig. 8G–J). Again, as negative controls, shared PMDs and HMDs failed to generate this distinguishing epigenetic pattern (Additional file 1: Fig. S7C).

These results prompted us to further investigate premalignant lesions, with the hypothesis that these methylation changes are pre-established in normal cells and preserved during the onset of neoplastic transformation. To address this, we interrogated public methylation data on BE, a recognized precursor to EAC, from two different studies [7, 8]. Importantly, the methylation patterns of BE samples were highly comparable with EAC tumors, showing reduced methylation levels in both EAC-specific PMDs and hypoDMRs in two different cohorts (Additional file 1: Fig. S7D-E). These data strongly suggest that epigenomic changes of PMDs and hypoDMRs occur in normal cells and are maintained in cancer, which further loses methylation in PMDs and gains additional DMRs. Moreover, these region-specific epigenomic regulations are shared across related cell types.

Encouraged by the above findings, we next sought to generate a classifier to predict cancer types based on their methylation levels of PMDs and DMRs. Specifically, we applied multinomial logistic regression models and used the leave-one-out cross-validation method to train the TCGA samples (*n* = 8915), which were categorized as either gastrointestinal, squamous, or other (neither gastrointestinal nor squamous) cancers, based on their methylation levels of PMDs and DMRs (See “ Methods”). Using the area under the Precision-Recall curve, we showed that both the PMDs and DMRs had high predictive performance, with the area under the curve (AUC) of PMDs ranging from 0.812 to 0.949 (Fig. 8K), and DMR from 0.836 to 0.958 (Fig. 8L). Combining PMD and DMR values (AUC ranging from 0.846 to 0.964) produced marginal improvement in performance (Fig. 8M), suggesting that the cell-of-origin information contained within PMDs is also captured within DMRs. These results were validated by the area under the receiver operating characteristic (ROC) curve (Additional file 1: Fig. S7F). Together, these data demonstrate that methylation levels of PMDs and DMRs are highly predictive of specific cell types and may serve as potential biomarkers for cancer diagnosis, especially when the two features are combined.

## Discussion

We generated one of the largest WGBS datasets in esophageal cancer to date, and here we focused on the analyses of PMDs (large scale) and DMRs (small scale) and revealed novel epigenomic properties of these regions. PMDs are megabase-long genomic regions with decreased DNA methylation, coinciding with heterochromatic late-replicating domains and Hi-C B domains [17]. PMDs reflect long-range chromatin organization that help orchestrate gene expression programs and can influence replication timing and 3D genome organization [24, 35, 62–64]. In addition, PMDs are associated with increased genomic instability and possibly activation of transposable elements (TEs) [19, 21]. Nevertheless, apart from these correlational observations, we have only limited mechanistic understanding of the origin and regulation of cancer PMD. Moreover, direct mechanisms linking PMDs to gene transcription remain to be established. Thus, a deeper characterization of PMD is warranted, which first requires an accurate and sensitive identification of these large domains from WGBS data. However, current PMD callers, including MethylSeekR and MethPipe, either are insensitive for the identification of shallow PMDs, or fail to call PMDs in tumor samples with extreme hypomethylation.

We have previously demonstrated that a local sequence context (solo-WCGW) is a strong determinant of DNA methylation loss at CpGs [19]. Extending this finding, we recently performed deep learning using the neural network method and established universal sequence context features influencing the hypomethylation of CpGs across the genome [34]. Here, we integrated this sequence code into the MethylSeekR program and developed a novel multi-model PMD caller, MMSeekR. Using both the Blueprint tumor WGBS dataset and our esophageal samples, we demonstrated a superior performance of MMSeekR over other current tools. In order to facilitate methodological development in the field of methylome investigation, we have made MMSeekR available at Github as a free software package (https://github.com/yuanzi2/MMSeekR).

The degree of variation of PMD methylation levels (depth) and genomic distribution (breadth) between cancer types was hitherto unclear. Here we observed strong heterogeneity at the PMD methylation level across cancer samples, while nonmalignant samples harbored expectedly shallow PMDs. Moreover, the genome fraction covered by PMDs varied profoundly among different samples, ranging from 24.3 to 63.4%. We identified and characterized subtype-specific PMDs, finding that they were associated with repressive transcription, B compartments, and high somatic mutation rate. We previously identified replication timing as a key determinant for methylation loss in PMDs [19]. However, this does not account for the variation in PMD genomic distribution across cell types. By investigation of the genome-wide occupancy of H3K36me2 in different cell types, we noted that H3K36me2 deposition correlated positively with HMD localization, while negatively with PMD in a cell-type-specific manner. Considering that H3K36me2 is able to recruit DNMT3A to maintain the level of DNA methylation [40], these results suggest that cell-type-specific deposition of H3K36me2 mark facilitates the maintenance of DNA methylation, thereby dictating the genomic distribution of HMDs and PMDs.

At a smaller genomic scale, we identified over ten thousand hypoDMRs between the two subtypes of esophageal cancer. Utilizing their matched nonmalignant samples, we further defined cell-type- *vs.* cancer-specific hypoDMRs. Using motif sequence analysis combined with ChIP-seq, we identified and validated candidate upstream regulators associated with either cell-type- or cancer-specific hypoDMRs. This approach is important for understanding of the transcriptional regulation during tumor development, particularly because increasing evidence has shown that tumor-driving transcription factors are often lineage-specific developmental regulators functionally co-opted to promote malignant cellular states [55, 56]. For example, our top candidate, HNF4A, is essential for the epithelial differentiation of the gastrointestinal tract. Consistently, we found that a substantial subset of cell-type-specific hypoDMRs contained HNF4A-binding sequence; these HNF4A^+^ cell-type-specific hypoDMRs were also co-enriched for transcript factors indispensable for normal gut development, such as FOXA1 (Fig. 6H). Importantly, compared with cell-type-specific hypoDMRs, HNF4A-binding sequence was significantly more enriched in tumor-specific hypoDMRs (Fig. 6H). Moreover, instead of FOXA1, these HNF4A^+^ tumor-specific hypoDMRs were co-enriched for AP-1 factors, which are well-recognized for their function in promoting EAC malignancy [59], similar to HNF4A itself [52, 53]. Consistently, one of the AP1 factors, FOSL1, has highly enriched binding sites in tumor-specific hypoDMRs as well as upregulated mRNA expression in EAC tumors relative to NGEJ. Importantly, we functionally validated that FOSL1 and HNF4A cooperatively bind to a subset of tumor-specific hypoDMRs. Together, careful dissection of cell-type- and cancer-specific hypoDMRs suggest that lineage master regulators control both normal and tumor cell transcriptomes, likely by occupying different genomic regions through cooperating with different transcriptional factor partners.

We further characterized the cell-type-specificity of PMDs and DMRs in normal cells. Starting from esophageal samples, we found that a large fraction of methylation changes in both PMDs and DMRs were already evident in normal samples. Pan-tissue single-cell ATAC-seq with 145,594 normal epithelial cells further showed that both PMDs and DMRs identified in esophageal cancer had strong specificity that was evident in related cell types. This was also observed in pan-cancer analyses of both methylation and ATAC-seq data from primary tumors, wherein cancers originating from related cell types exhibited similar profiles of both PMDs and DMRs. Moreover, by measuring cancer precursor lesions, we demonstrated that epigenomic changes of PMDs and DMRs were preserved during the onset of neoplastic transformation. Nonetheless, PMDs in normal samples were much shallower than tumors (Fig. 2A and C *vs*. Fig. 7A).

## Conclusions

This study highlights the presence of cell-type-specific PMDs and DMRs in normal cell types, which are preserved in malignant cells. To our knowledge, this is the first demonstration of the prominent cell-type specificity of PMDs across normal, precursor, and malignant states. While prior studies have revealed that DMRs contain tissue-specific regulatory regions, here we present a paradigm for distinguishing cell-type- vs. cancer-specific regions, and use those to identify tumor-specific regulatory mechanisms.

## Methods

### Cell culture

ESCC cell lines (TE5 and KYSE70) and EAC cell lines (OE19 and ESO26) were kindly provided by Dr. Koji Kono from Cancer Science Institute of Singapore, and Dr. Stephen Meltzer from Johns Hopkins University, respectively. These cell lines were authenticated by the short tandem repeat analysis and were tested negative for mycoplasma. They were grown in RPMI-1640 medium (Gibco, USA), supplemented with 10% FBS (Omega Scientific, USA) and 1% penicillin–streptomycin (Thermo Scientific, USA). All cultures were maintained in a 37 °C incubator supplemented with 5% CO_2_.

### Whole-genome bisulfite sequencing (WGBS)

WGBS of ESO26 or TE5 cells was performed at Novogene, Inc. Briefly, after DNA extraction and quality control (QC), 3 µg DNA of ESO26 or TE5 cells spiked with 26 ng lambda DNA were fragmented by sonication. The sonicated DNA was ligated with different cytosine-methylated molecular barcodes. Next, bisulfite conversion was performed using EZ DNA Methylation-GoldTM Kit (Zymo Research). PCR amplification with KAPA HiFi HotStart Uracil + Ready Mix (Kapa Biosystems) was then applied to the DNA fragments. The clustering of index-coded DNA samples was sequenced using the Illumina Hiseq 2500 platform.

### Chromatin immunoprecipitation sequencing (ChIP-Seq) and ChIP-qPCR

Ten million esophageal cancer cells were harvested and transferred into 15 ml tubes, followed by fixing with 4 ml of 1% paraformaldehyde for 10 min under room temperature. The reaction was stopped by 2 ml of 250 mM of glycine. Cell samples were rinsed twice by 1 × PBS and lysed by 1 ml of 1 × lysis/wash buffer (150 mM NaCl, 0.5 M EDTA pH 7.5, 1 M Tris pH 7.5, 0.5% NP-40). Cell pellets were next resuspended using shearing buffer (1% SDS, 10 mM EDTA pH 8.0, 50 nM Tris pH 8.0) followed by sonication using a Covaris sonicator. Subsequently, debris was removed by centrifuge and supernatants were diluted five times with the buffer containing 0.01% SDS, 1% Triton X-100, 1.2 mM EDTA pH 8.0, 150 nM NaCl. One microgram of indicated antibodies (H3K36me2, Cell Signaling Technology, # 2901S; FRA1, Cell Signaling Technology, #5281) [65, 66] was added and incubated by rotation at 4℃ overnight. Protein G Dynabeads (Life Technologies, USA) were added the next morning and incubated by rotation for an additional 4 h. Dynabeads were next washed with 1 × wash buffer followed by cold TE buffer. DNAs were reverse crosslinked, purified, followed by library preparation and deep sequencing using the Illumina HiSeq platform.

For shRNA knockdown of HNF4A in ESO26 and OE19 cells, we used the procedures published by us previously [53], using the pLKO-puro vector (Addgene, #8453) containing shRNA sequence: CCGGACATCAACGACCGCCAGTATGCTCGAGCATACTGGCGGTCGTTGATGTTTTTTGAATT (5′ to 3′).

### Data sources

DNA methylome of esophageal samples were obtained from our recent work [27], including WGBS on 21 ESCC, 3 NESQ, 5 EAC, 7 GEJ tumors, and 7 NGEJ tissues [67]. We obtained additional two NESQ samples from the ENCODE consortium to ensure statistical power. Considering the indistinguishable clinical and molecular characteristics between EAC and GEJ tumors, in the present study they were combined as the same subtype (referred to as EAC), which is a common strategy in the field [3]. TCGA Pan-cancer DNA methylome derived from HM450k methylation array was downloaded from GDC v16.0 by TCGAbiolinks package (version 2.13.6) [68]. Other DNA methylation data from individual studies, including EAC EPIC array data from the Oesophageal Cancer Clinical and Molecular Stratification (OCCAMS) consortium (EGAD00010001822) [69], EAC and BE methylome from GSE72874 [70] and GSE81334 [71], along with ESCC tumor WGBS data (GSE149608 and PRJNA523898) [72, 73], were analyzed for validation purposes in this study.

Other public datasets which were analyzed included bulk ATAC-seq data of pan-cancer samples from TCGA [74], single-cell ATAC-seq data across different adult human tissues (GSE184462) [75], H3K27ac ChIP-seq in EAC samples (GSE132680) [76], EAC cell lines (ESO26, FLO1, JH-EsoAd1, OACp4C, OE19, OE33, SKGT4 from GSE132680) [76], and ESCC cell lines (KYSE140, KYSE70, TE5 from GSE106563 [77]; KYSE150, KYSE180, KYSE200 from GSE131490 [78]; TE7 from GSE106433 [79]), HNF4A ChIP-seq in OE19 (E-MTAB-6858) [80] and ESO26 cell lines (GSE132813) [81], GATA4 ChIP-seq in ESO26 cell line (GSE132813) [81] and TP63 ChIP-seq in TE5 cell line (GSE148920) [82]. H3K36me2 ChIP-seq of wildtype (NSD1-WT) HNSCC cell lines were downloaded from GSE149670 [83]. Somatic mutation datasets were downloaded from individual studies [9, 84]. We also retrieved the transcriptomic data of esophageal cancer from the TCGA consortium [85] and GSE149609 [86]. CGI promoters are annotated as regions ranging from 250 bp upstream to 500 bp downstream of any TSSs overlapping with Takai CGIs [26]. Repetitive elements, including long interspersed nuclear elements (LINE), short interspersed nuclear elements (SINE), and long terminal repeats (LTR), were extracted from UCSC website [87]. We downloaded the annotation of common PMDs (defined as shared PMDs identified from 40 different cancer types) as well as solo-WCGW [88] and ENCODE blacklist regions [89]. All of the annotations were converted to the hg38 version using the UCSC LiftOver script (https://genome.ucsc.edu/cgi-bin/hgLiftOver). The human core transcription-factor-binding sequences in the HOMOCOMO database (version 11) were used for motif annotation [90].

### DNA methylation data analysis

For WGBS data, raw reads were mapped to the human genome (GRCh38) by Biscuit align command (version 0.1.4, https://www.githubcom/zwdzwd/biscuit) with default settings. Mapped reads were sorted by genome position, and duplicates were marked using Picard MarkDuplicates tool (version 1.136, http://broadinstitute.github.io/picard/). Biscuit pileup and vcf2bed command were then used to extract DNA methylation information. All CpG sites with a coverage ≥ 3 informative reads and outside of the ENCODE blacklist regions were retained for downstream analyses. For EPIC and HM450K array data, methylation of each probe was extracted using the SeSAME package with noob and dyeBiasCorrTypeINorm function for background subtraction and dye bias correction [91]. To calculate the mean methylation levels within shared PMDs/HMDs, EAC-specific PMDs and ESCC-specific PMDs, solo-WCGW CpG probes on EPIC and HM450K arrays were used. According to the annotation of Infinium DNA methylation arrays [92], recommended general masking probes were removed. HM450K methylation data from the TCGA were used to estimate the chromatin A/B compartments using minfi compartments function with “resolution = 100*1000, what = OpenSea” options [35]. Briefly, ~ 170,000 open sea probes on the HM450k array showed the strongest correlation with A/B compartments and were used in the prediction process. A *p* x *n* methylation matrix was generated for each chromosome, where *p* refers to the normalized probes and *n* represents the samples. Next, we calculated the correlation between pairwise probes and obtained the *p* x *p* correlation matrix. Then the correlation matrix was grouped into bins based on a predetermined resolution *k* and the median correlation between the CpGs contained in each bin was calculated. Bins without any probes were removed.

### Development of a sequence-aware PMD calling method: multi-model PMD SeekR (MMSeekR)

We recently performed neural network-based machine learning to establish local DNA sequence features of CpGs that were associated with global DNA methylation loss, and derived a neural network (NN) score for each CpG across the human genome [34]. In order to exclude the potential impact of high CpG density (such as CpG island), we reserved CpGs having 2 or fewer neighboring CpGs within the 151-bp window centered on the reference CpG. We investigated the correlation between NN scores and methylation in individual samples in non-overlapping 201-CpG windows across the genome. As expected, due to the greater degree of methylation loss within PMDs, there was a strong negative correlation between DNA methylation levels and NN scores within windows in PMDs, in contrast to much more modest correlations within highly methylated domains (HMD) windows (Additional file 1: Fig. S2A).

We next applied Pearson correlation coefficient (PCC) between our NN score and DNA methylation, as well as the “alpha score” used in the MethylSeekR model, to 201-CpG windows genome-wide. Compared with the NN score, the MethylSeekR alpha score is a very different measurement, returning a high score if the distribution of methylation values is closer to a unimodal beta distribution centered on 0.5 (typical of PMDs) than it is to a bimodal methylation value distribution close to 0 and 1 (typical of HMDs). Specifically, we applied a Hidden Markov Model (HMM) segmentation (as in MethylSeekR) to each model independently and found that both the PCC and MethylSeekR alpha score showed bimodal distributions for the testing sample (Additional file 1: Fig. S2B-C). We hypothesized that since the PCC and the alpha score were very different models, combining them might improve the performance of PMD calling (Additional file 1: Fig. S2D). Thus we developed a “2-dimensional (2D)” model accordingly (Fig. 1C). This 2D model performed comparably well or better than either MethylSeekR or MethPipe in most cases, returning results consistently and highly overlapping with common PMDs (Additional file 2: Table S2).

While the 2D model generally performed well, we did note that it failed in a few samples with extreme methylation loss. Interestingly, these failed cases universally showed PMD methylation values very close to 0, which would be expected to violate the assumptions of both the PCC model and alpha model due to lack of variance within PMDs (Fig. 1C right part). We thus postulated the raw methylation values (transformed to an M-value to disperse scores close to 0 and 1) might provide additional predictive power in certain samples with extreme methylation loss, and we developed a 3D model accordingly by adding the M-value model to the 2D model. In order to decide whether the 2D or 3D model should be applied for any given sample, we first measured the methylation values of all CpGs with 2 or fewer neighboring CpGs within a 151-bp window, which excludes most CpG islands and contains a set of CpGs that are strongly associated with PMD hypomethylation [19]. If the bottom 10th percentile of these CpGs had a methylation value below 0.025, the 3D model was selected; otherwise, the 2D model was selected. This was based on the observation that the majority of samples with extreme methylation loss failed under both the MethylSeekR and MMSeekR 2D model (Fig. 1C).

### Application of MMSeekR to WGBS data

MMSeekR was applied to call PMDs in each WGBS sample. Before PMD calling, CpG sites with coverage of fewer than 5 informative reads were excluded. Then ENCODE blacklist regions were subtracted from the resulting PMDs. Within each esophageal cancer subtype, PMDs generated from each sample were integrated using bedtools multiinter function (version 2.27.1, https://bedtools.readthedocs.io/en/latest/). The common PMD set for each subtype contained those occurring in at least two-thirds of samples from that subtype. We further defined subtype-specific PMDs as those common PMDs from one subtype that were detected in fewer than one-third of samples in the other subtype. Meanwhile, PMDs that were in both the common EAC set and the common ESCC set were denoted as shared PMDs. Regions that were PMDs in < 1/3 samples of both subtypes were denoted as shared HMDs.

### Identification and characterization of DMRs

Regions belonging to either the common ESCC or common EAC PMD sets were masked out from the DMR analysis. The Dmrseq method [93] has been widely used for DMR calling, albeit with its own limitations, including large CPU requirements and some of the long DMR regions identified. We used Dmrseq package (version 1.10.0) to identify DMRs between ESCC and EAC tumors with the following parameters: cutoff = 0.1, bpSpan = 1000, minInSpan = 30, maxPerms = 500. Since the coverage information of each CpG site is required by dmrseq for statistical inference, here we included all CpG sites with ≥ 3 informative reads. Regions with *q* value < 0.05 and absolute delta methylation change > 0.2 were identified as DMRs. For hypomethylated DMRs (hypoDMRs) from each cancer subtype, we further performed one-tailed *t*-tests comparing the mean methylation within the DMR in nonmalignant *vs.* tumor samples, and those with FDR < 0.1 were considered as tumor-specific (ts)-hypoDMRs. Both hypoDMRs and ts-hypoDMRs were annotated using HOMER annotatePeaks.pl script (version 4.9.1) [50].

### Calculation of mean DNA methylation levels

CpG sites with a coverage of at least 5 informative reads were used for this calculation. Average methylation levels of CpG sites across the genome (global level), within CGI promoters, commonPMDs, SINE, LINE, and LTR in each sample were calculated independently. Besides, we obtained the mean methylation of CpG sites in non-PMD regions. For genome/domain-wide visualization, the average methylation of 10-kb consecutive non-overlapping tiles was shown. To calculate the mean methylation levels within shared PMDs/HMDs, EAC-specific PMDs, and ESCC-specific PMDs, solo-WCGW CpG sites/probes were used.

### Principal component analysis of WGBS data

PMDs were identified by either MethPipe, MethylSeekR, or MMseekR (Fig. 1D). The whole genome was split into 30-kb consecutive but non-overlapping tiles. For each tile, the ratio overlapping with any PMD was calculated for each caller. The top 5000 most variable 30-kb tiles from each PMD caller were used in principal component analysis (PCA). In Additional file 1: Fig. S4A and S4C, CpG sites with at least 7 reads across all esophageal samples were used. Then the top 8000 most variable CpG sites were selected for PCA using the R prcomp function. PCA was performed before and after masking the combined common PMDs from EAC and ESCC and generated the point plots by ggplot2 package (version 3.1.0).

### RNA-seq data analysis

According to the raw read counts obtained from the TCGA, we identified significant upregulated genes by DESeq2 package (version 1.22.2) with adjusted *p*-value < 0.05, fold change > 2, and mean FPKM > 0.1 in the corresponding sample groups [94]. For expression datasets of nonmalignant squamous and ESCC tissues, raw reads were aligned to GRCh38 using HISAT2 (version 2.0.4) [95] and quantified by htseq-count program (version 0.11.2) at default setting. Significant upregulated genes were identified using the same method as for the TCGA datasets.

### ChIP-seq data analysis

Raw reads were mapped to GRCh38 (ENSEMBL release 84) using BWA mem program (version 0.7.15) with the default options [96]. Then the mapped reads were sorted using SAMtools program (version 1.3.1) [97], followed by removing PCR duplicates and blacklist regions by Picard MarkDuplicates tool and bedtools (version 2.27.1). MACS2 (Model-Based Analysis of ChIP-Seq, version 2.1.2) were applied to call peaks with the default setting for transcription factors, ''-q 0.01–extsize = 146 –nomodel'' options for H3K27ac and ''–broad -p 0.01 –extsize = 146 –nomodel'' for H3K36me2 [98]. Bigwig files were generated by deepTools bamCompare function (version 3.1.3) with “–operation subtract –normalizeUsing CPM –extendReads 146 –binSize 20” parameters [99]. Average signals of shared PMDs/HMDs, EAC-only PMDs, and ESCC-only PMDs in each H3K27ac or H3K36me2 ChIP-seq sample were extracted from bigwig files using deepTools computeMatrix function with ''scale-regions'' option.

### ATAC-seq data analysis

For bulk pan-cancer ATAC-seq data obtained from the TCGA project, the average accessibility of regions/domains was extracted from the available bigwig files using deepTools computeMatrix function [42]. To avoid the influence of scaling factors across different samples and batches, the mean accessibility across the whole genome in each sample was calculated and used for normalization. For single-cell ATAC-seq data, based on the clustering and annotation results from the publication [60], only epithelial cell types were used for further analysis. Similarly, the average accessibility of regions/domains was derived for each cell in each sample and normalized by the mean signal across the whole genome.

### DMR motif enrichment analysis

For each hypoDMR or ts-hypoDMR, we randomly sampled 10 regions with the same size and number of CpGs to define the background set. Then motif searching of both DMRs and background regions was performed using HOMER annotatePeaks.pl with ''-noann -m HOCOMOCOv11_core_HUMAN_mono_homer_format_0.0001.motif'' parameters [50]. The ELMER method was next applied to identify potential transcription-factor-binding sequences and the top 15 transcription factors with *q* value < 0.05 and FPKM > 5 in the corresponding cancer subtype were reserved for further analysis [47].

### Pathway enrichment analysis

We performed the pathway (Biological Process) enrichment analysis by Cistrome-GO [100] using candidate regions with methylation changes and differential expression analysis results. For hypoDMR analysis, subtype-specific DMRs and upregulated genes in the corresponding tumors were used as input data. For subtype-specific PMDs, the input data contained PMD regions and downregulated genes in the corresponding tumors. The top 15 enriched pathways with *q* value < 0.05 were shown.

### A cancer type classifier based on the methylation levels of PMDs and DMRs

We collected all TCGA samples (*n* = 8915), which were categorized as either gastrointestinal (*n* = 875), squamous (1370), or other (neither gastrointestinal nor squamous, *n* = 6670) cancers, and calculated their methylation levels of subtype-specific PMDs and DMRs. Due to the sample size bias, we performed sample downsizing and randomly selected 20% samples of other cancers (*n* = 1334) to achieve a balanced training set. Then we applied multinomial logistic regression models with the “multiROC” package and used the leave-one-out cross-validation method for data training. The mean methylation values of subtype-specific PMDs or DMRs in each sample were used as the input variables for PMD or DMR model training, respectively. To train the combined model, subtype-specific PMDs and DMRs were used together as input variables. This process was repeated 100 times and the training results were merged when plotting the Precision-Recall and ROC curves.

### Supplementary Information

**Additional file 1: Figure S1. **Methylation landscape of 45 esophageal WGBS samples. **Figure S2. **The development of MMSeekR, a sequence-aware multi-model PMD caller. **Figure S3. **Analyses of subtype-specific PMDs. **Figure S4. **DMR analyses upon masking of union PMDs. **Figure S5. **Characterization of tumor-specific hypoDMRs. **Figure S6. **Characterization of tumor-specific hyperDMRs. **Figure S7. **Neither shared PMDs nor HMDs show cell-type specificity.

**Additional file 2: Table S1. **WGBS data sets used in the current study.** Table S2. **F1-socres for three different callers in each tumor sample from the Blueprint consortium. **Table S3.** F1-socres for three different callers  in each esophageal tissue.

**Additional file 3. **Review History.

## Data Availability

WGBS data and ChIP-seq data for H3K36me2 in EAC and ESCC cell lines were available at GSE210220 [101]. Source code for MMSeekR is available under the MIT license at Github https://github.com/yuanzi2/MMSeekR [102] and at Zenodo 10.5281/zenodo.8210135 [103]. Source code for WGBS data analysis and figure reproduction is under the MIT license at Github https://github.com/yuanzi2/ESCA_WGBS_analysis [104] and at Zenodo 10.5281/zenodo.8210149 [105]. The methylation bed files are available at https://zenodo.org/record/6954946 [67]. Other datasets used in this study were downloaded from the following links: EAC EPIC array: EGAD00010001822 [69]; EAC and BE methylome: GSE72874 [70] and GSE81334 [71]; ESCC tumor WGBS data: GSE149608 [72] and PRJNA523898 [73]; TCGA bulk ATAC-seq data: GDC [74]; single-cell ATAC-seq data across different adult human tissues: GSE184462 [75]; H3K27ac ChIP-seq of EAC samples: GSE132680 [76]; H3K27ac ChIP-seq of EAC cell lines: ESO26, FLO1, JH-EsoAd1, OACp4C, OE19, OE33, SKGT4 from GSE132680 [76]; H3K27ac ChIP-seq of ESCC cell lines: KYSE140, KYSE70, TE5 from GSE106563 [77]; KYSE150, KYSE180, KYSE200 from GSE131490 [78]; TE7 from GSE106433 [75]; HNF4A ChIP-seq: OE19 from E-MTAB-6858 [80]; ESO26 from GSE132813 [81]; GATA4 ChIP-seq: ESO26 from GSE132813 [81]; TP63 ChIP-seq: TE5 from GSE148920 [82]; H3K36me2 ChIP-seq: wildtype (NSD1-WT) HNSCC cell lines from GSE149670 [83]; Somatic mutation datasets were downloaded from supplementary files in individual studies [9, 84]; mRNA expression of esophageal cancer: TCGA [85] and GSE149609 [86]; CGI promoters: Takai CGIs [26]; Masked CGI regions [39]; Repetitive elements: LINE, SINE, and LTR [87]; PMDs as well as solo-WCGWs [88].
