# Supplementary material for: Comprehensive analyses of partially methylated domains and differentially methylated regions in esophageal cancer reveal both cell-type- and cancer-specific epigenetic regulation
Source: Genome Biol. 2023 Aug 24;24:193. doi: 10.1186/s13059-023-03035-3 (PMC10463844; doi:10.1186/s13059-023-03035-3)
Supplement: Supplementary file 1 — Additional file 1: Figure S1. Methylation landscape of 45 esophageal WGBS samples. Figure S2. The development of MMSeekR, a sequence-aware multi-model PMD caller. Figure S3. Analyses of subtype-specific PMDs. Figure S4. DMR analyses upon masking of union PMDs. Figure S5. Characterization of tumor-specific hypoDMRs. Figure S6. Characterization of tumor-specific hyperDMRs. Figure S7. Neither shared PMDs nor HMDs show cell-type specificity. [file 13059_2023_3035_MOESM1_ESM.docx]

**Supplementary Figures**


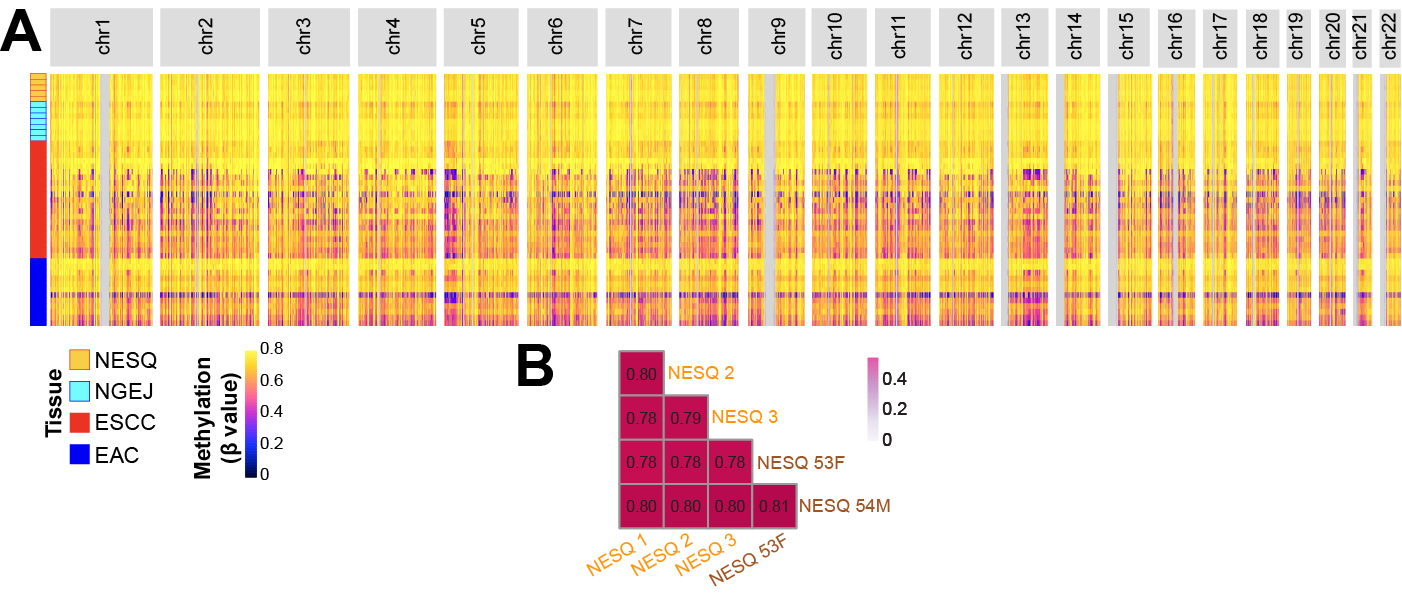


**Figure S1. Methylation landscape of 45 esophageal WGBS samples. (A)** Genome-wide maps of DNA methylation profiles from 45 esophageal WGBS samples. Average methylation values were shown in consecutive and non-overlapping 10-kb tiles. CGI regions were masked using the annotation from Irizarry et al. **(B)** NESQ tissues show high inter-sample correlation in DNA methylation levels despite from two different datasets. NESQ1, NESQ2 and NESQ3 are internal samples while the other two are from ENCODE projects.


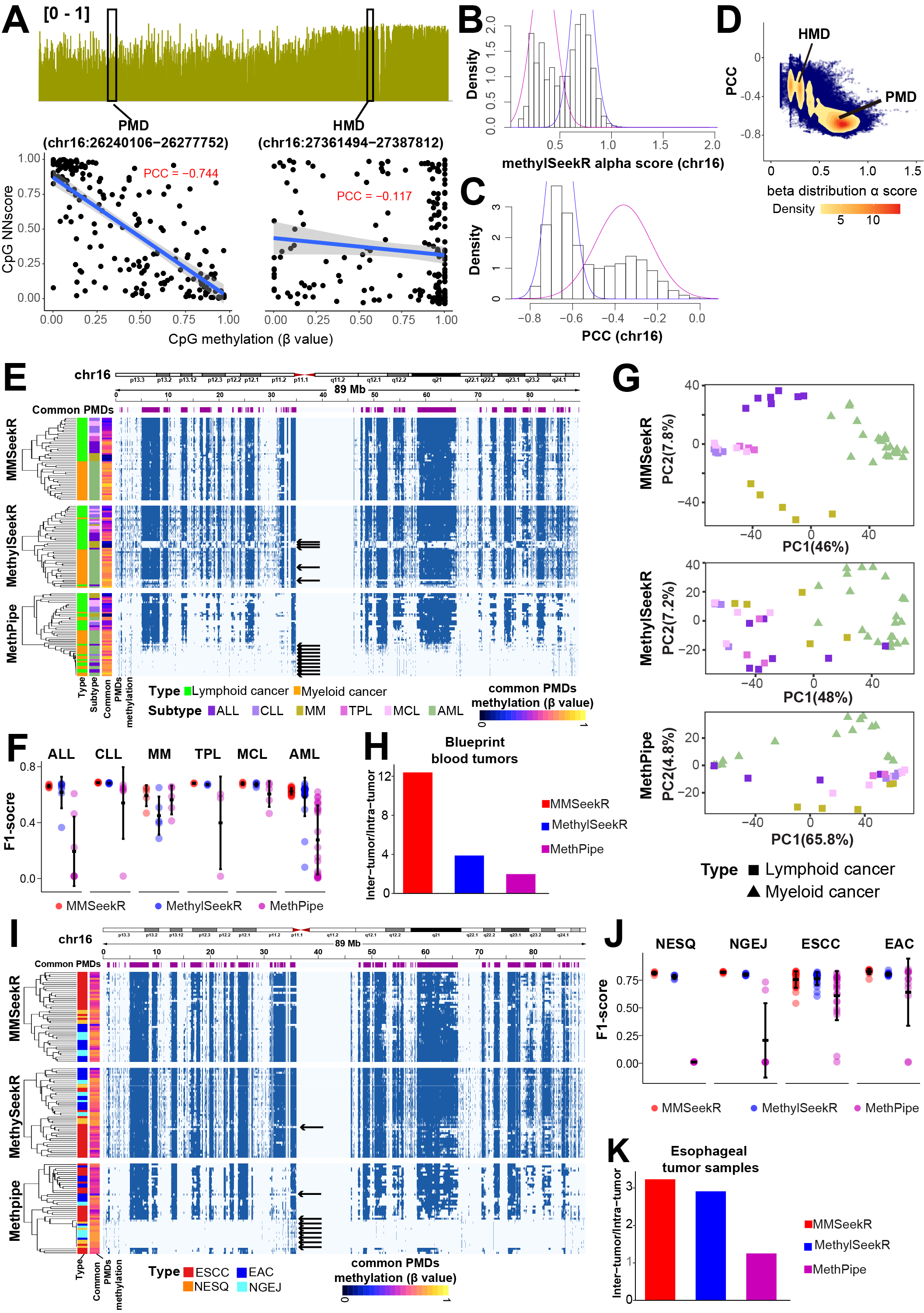


**Figure S2. The** **development of MMSeekR, a sequence-aware multi-model PMD caller.** **(A)** Pearson correlation coefficients between NN scores and DNA methylation levels in individual CpGs in representative PMD or HMD windows. **(B)** MethylSeekR α scores and (**C**) Pearson correlation coefficient across all 201-CpG windows of chr16 show bimodal distributions. **(D)** A density plot of the distributions of Pearson correlation coefficient and MethylSeekR α scores. The methylome data in **(A-D)** are from a Blueprint tumor sample “S01FJZA1_MantleCellLymphoma”. **(E)** PMD regions (purple) across chr16 identified by the three PMD callers; the 47 Blueprint samples were clustered by the PMD distribution across the genome. **(F)** F1-score comparison of different PMD callers for each Blueprint blood tumor group. F1-score is the harmonic mean of precision and recall; precision was computed as the ratio of the intersection size between predicted PMDs and common PMDs over the total size of predicted PMDs; recall was computed as the ratio of the intersection size between predicted PMDs and common PMDs over the total size of common PMDs. **(G)** PCA analysis using the top 5,000 most variable 30-kb tiles from the three PMD callers. Data used in this figure are from Blueprint consortium. **(H)** The average PCA distance ratio of inter-tumor versus intra-tumor samples for each PMD caller using our esophageal tumor samples. **(I-K)** Similar to (**E, F and H)**, showing PMD regions (blue) across chr16 identified by the three PMD callers **(I)** and their prediction performance **(J-K)**; esophageal samples were clustered by PMD distribution across the genome. Mean and standard deviation in **(F, J)** are indicated by the center ticks and error bars, respectively. Black arrows in **(E, I)** point to failures in MethylSeekR and Methpipe methods.


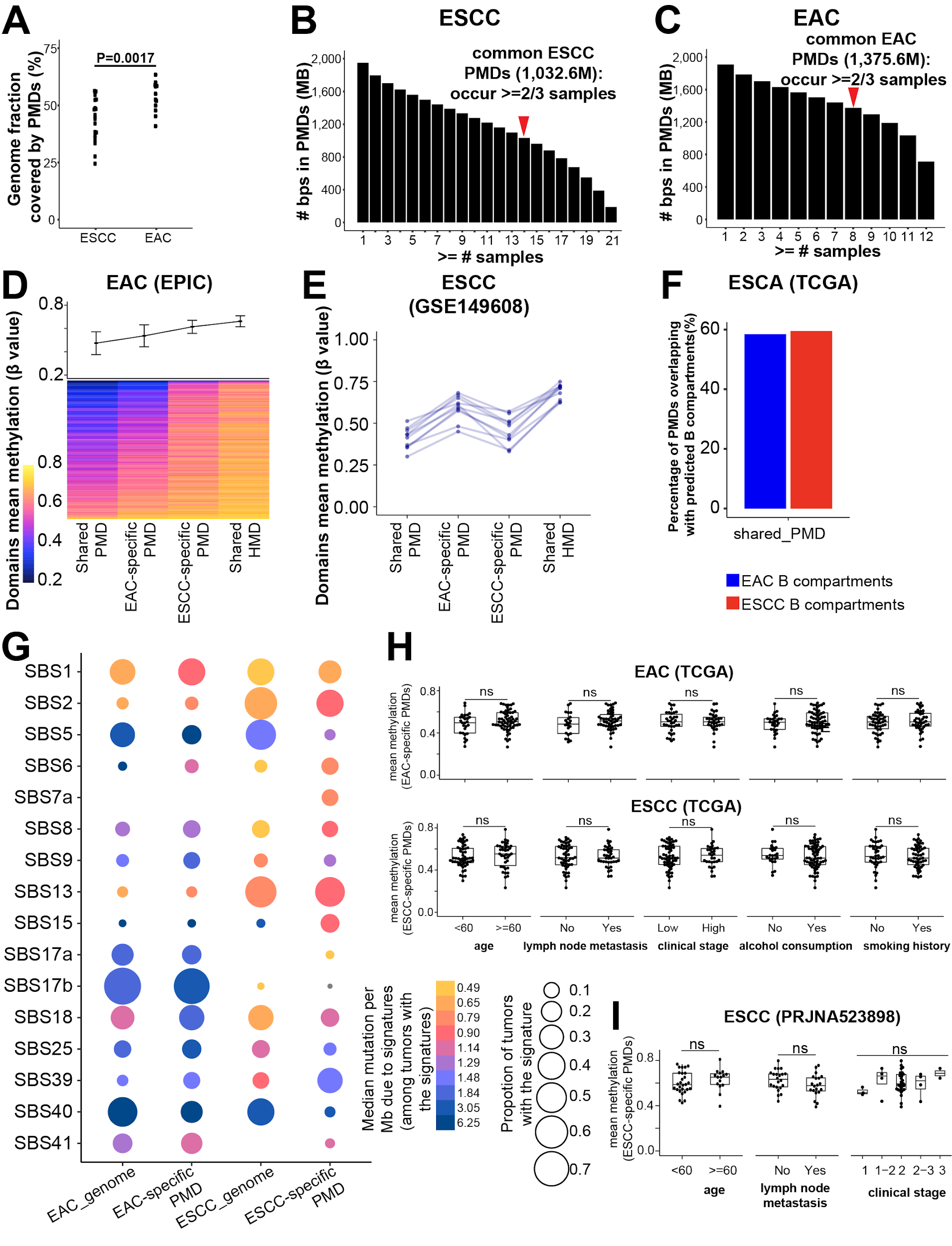


**Figure S3. Analyses of subtype-specific PMDs. (A)** Genome fractions covered by PMDs in each tumor sample. The P value was determined by a two-tail t test. **(B-C)** Genomic regions covered by PMDs which are common in ESCC **(B)** or EAC samples **(C)**. **(D)** Heatmaps and line plots displaying the methylation levels for different PMD categories in EAC. Each row in the heatmap shows methylation (beta value) per sample; the trend line displays the average methylation and standard deviation. EPIC arrays data were obtained from the Oesophageal Cancer Clinical and Molecular Stratification (OCCAMS) consortium (EGAD00010001822), containing 292 EAC samples. **(E)** Line plots showing the average methylation levels for different PMD categories in ESCC. Each line represents one sample. WGBS data are from GSE149608, including 9 ESCC samples. **(F)** Bar plots showing the percentage of shared PMDs overlapping with chromatin B compartments, which were defined by TCGA methylation datasets using minfi package. **(G**) Mutational signatures across the whole genome or ts-specific PMD regions in either EAC or ESCC tumors. The dot size denotes the proportion of samples exhibiting the indicated mutational signature. The dot color represents the median mutational burden of the corresponding mutational signature. Mutational signatures with the sample proportion > 0.1 in any one group are shown. **(H)** Boxplots showing the mean methylation of subtype-specific PMDs in TCGA EAC (n=87) or ESCC samples (n =90), calculated separately for each category of clinical features. DNA methylation values in TCGA samples were extracted from the HM450k array. **﻿(I)** The mean methylation of ESCC-specific PMDs were calculated using a published ESCC WGBS dataset (PRJNA523898, n=42). ﻿For panel **H**, a two-tailed t-test was performed for each comparison pair. For panel **I**, a two-tailed t-test was performed for age and lymph node metastasis, and a two-way ANOVA was performed for TNM_stage.


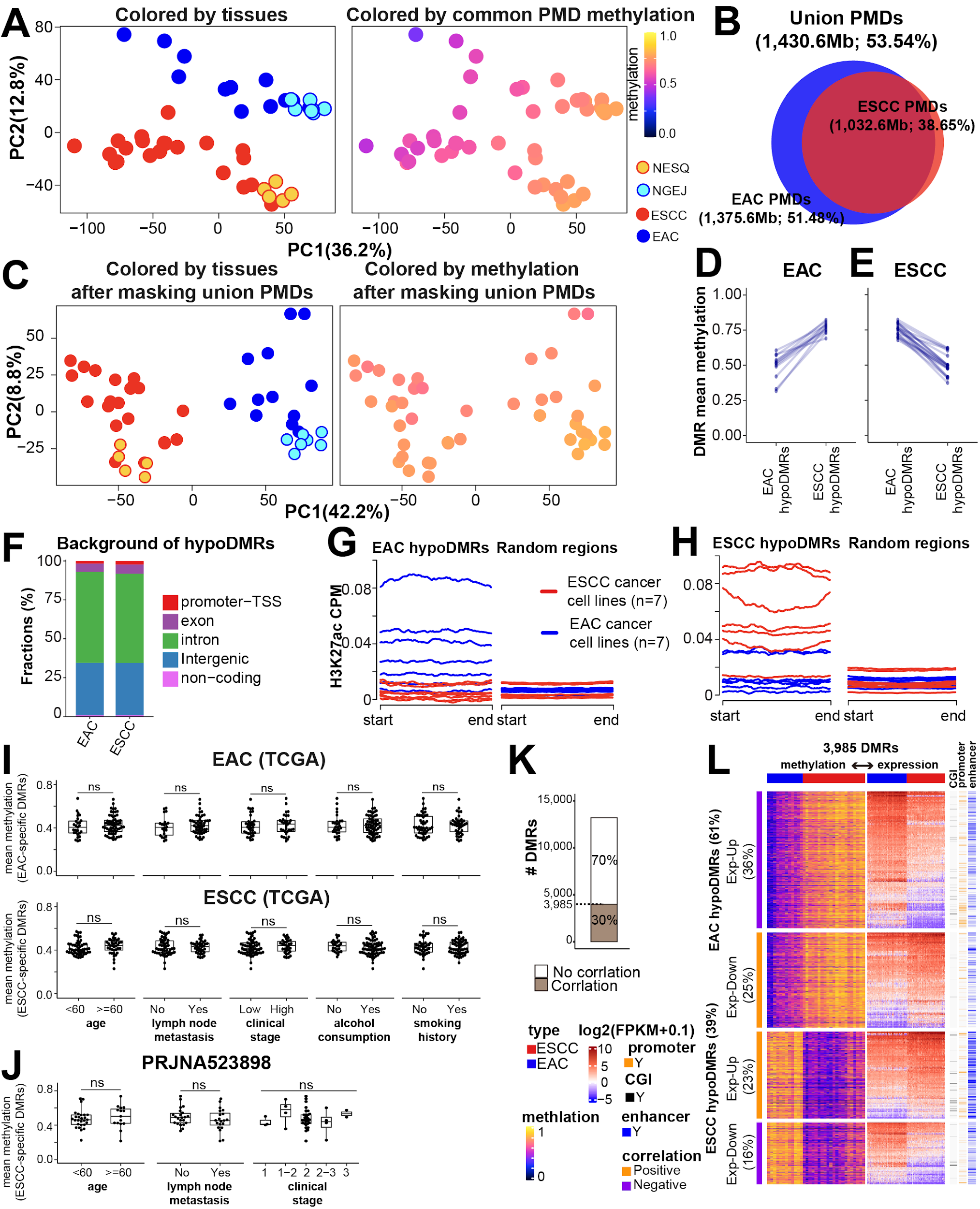


**Figure S4. DMR analyses upon masking of union PMDs. (A)** PCA analysis using the top 8,000 most variable CpGs with a coverage higher than 7 in each sample. The samples are colored by tissue types (left) and methylation levels of common PMDs (right). **(B)** A Venn diagram showing the union PMD set combining EAC and ESCC PMDs, which were identified according to **Fig. S2B-C**. **(C)** PCA analysis upon masking the union PMD set, again using the top 8,000 most variable CpG sites with a coverage higher than 7. **(D-E)** Line plots showing the average methylation levels for different hypoDMR categories in EAC **(D)** and ESCC **(E)**. Each line represents one sample. **(F)** Stacked bar plots showing fractions of hypoDMRs that overlap with different genomic features. Random genomic regions contained 10-times randomly selected regions with the same CpG density. **(G-H)** Aggregation line plots showing H3K27ac ChIP-seq signals in either EAC **(G)** or ESCC **(H)** hypoDMRs from esophageal cancer cell lines. **(I)** Boxplots showing the mean methylation of subtype-specific DMRs in TCGA EAC (n=87) or ESCC samples (n =90), calculated separately for each category of clinical features. DNA methylation values in TCGA samples were extracted from the HM450k array. **﻿(J)** The mean methylation of ESCC-specific DMRs were calculated using a published ESCC WGBS dataset (n=42). ﻿For panels **I**, a two-tailed t-test was performed for each comparison pair. For panel **J**, a two-tailed t-test was performed for age and lymph node metastasis, and a two-way ANOVA was performed for clinical stage. **(K)** Number of DMRs correlated with gene expression levels. **(L)** Heatmaps showing DMRs significantly correlated with gene expression levels. The left heatmap shows the methylation level (beta value) per DMR per sample; the right heatmap displays the mRNA level of the closest gene to the DMR per sample (z-scored). DMRs overlapping with different genomic features were shown. Methylation values were extracted from the WGBS dataset. Expression data were obtained from the TCGA project.


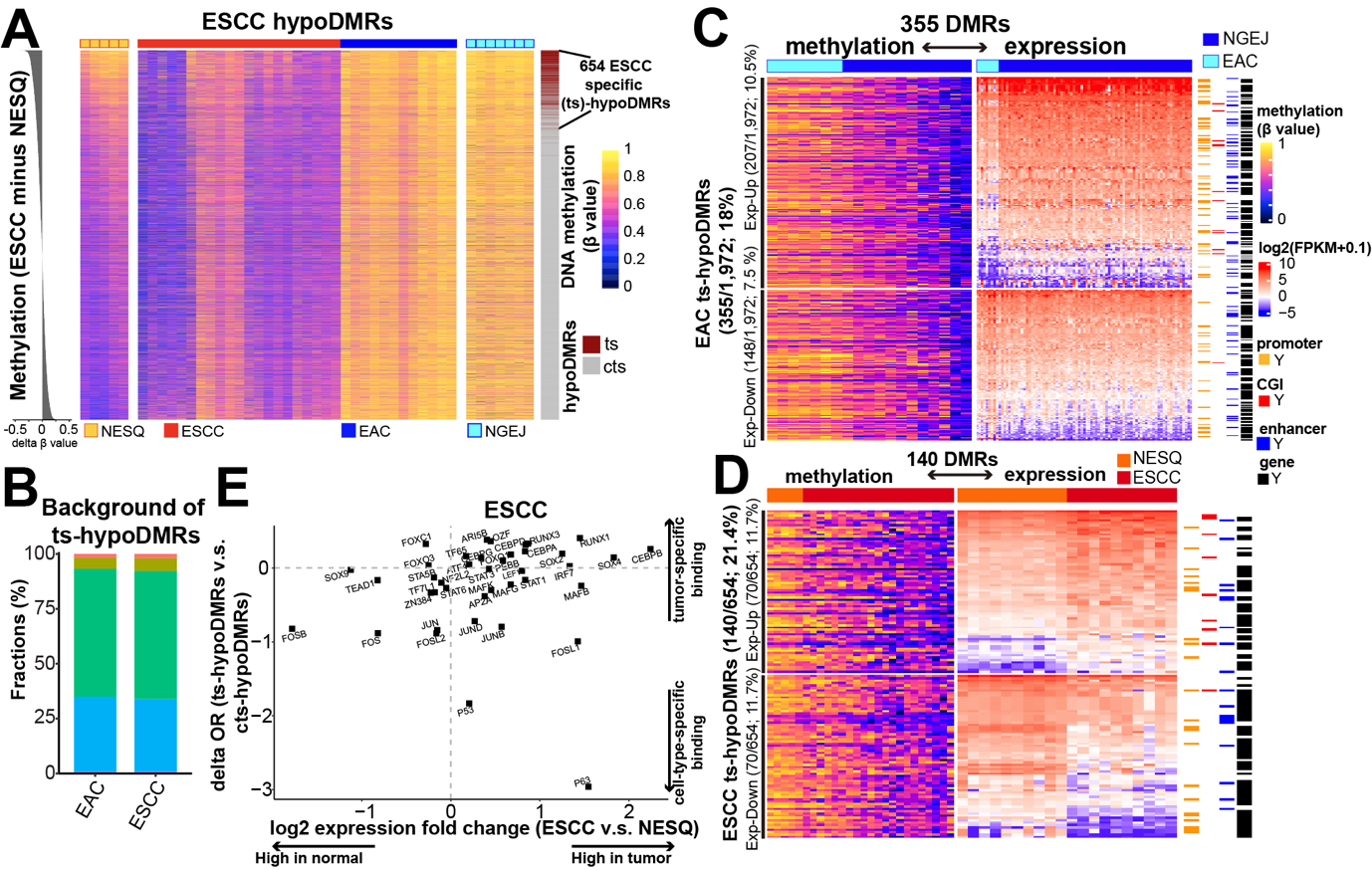


**Figure S5. Characterization of tumor-specific hypoDMRs. (A)** Similar to **Fig. 6A**, heatmaps showing methylation levels for each ESCC hypoDMR. 654 ESCC ts-hypoDMRs were identified using one-tailed t test between ESCC and NESQ samples (right) with the FDR cutoff < 0.05. **(B)** Stacked bar plots showing fractions of ts-hypoDMRs associated with random regions that overlap with different genomic features. **(C-D)** Heatmaps showing EAC **(C)** or ESCC **(D)** ts-hypoDMRs correlated with gene expression levels. The left heatmap shows the methylation level (beta value) per DMR per sample; the right heatmap shows the mRNA expression level of the closest gene to the DMR per sample (z-scored). DMRs overlapping with different genomic features are shown. Methylation values were extracted from the WGBS dataset. The expression of EAC and paired nonmalignant tissues were obtained from the TCGA project, while the ESCC expression data were from GSE149609. **(E)** Scatter plots showing the transcription-factor-binding sites that were enriched in ESCC ts-hypoDMRs compared with cts-hypoDMRs. The X axis represents the expression fold change between ESCC and paired NESQ samples. The Y axis shows the delta enrichment score of transcription-factor-binding sites between ts- *vs.* cts-hypoDMRs. Expression data were from the GSE149609 and motif enrichment analyses were performed by ELMER.


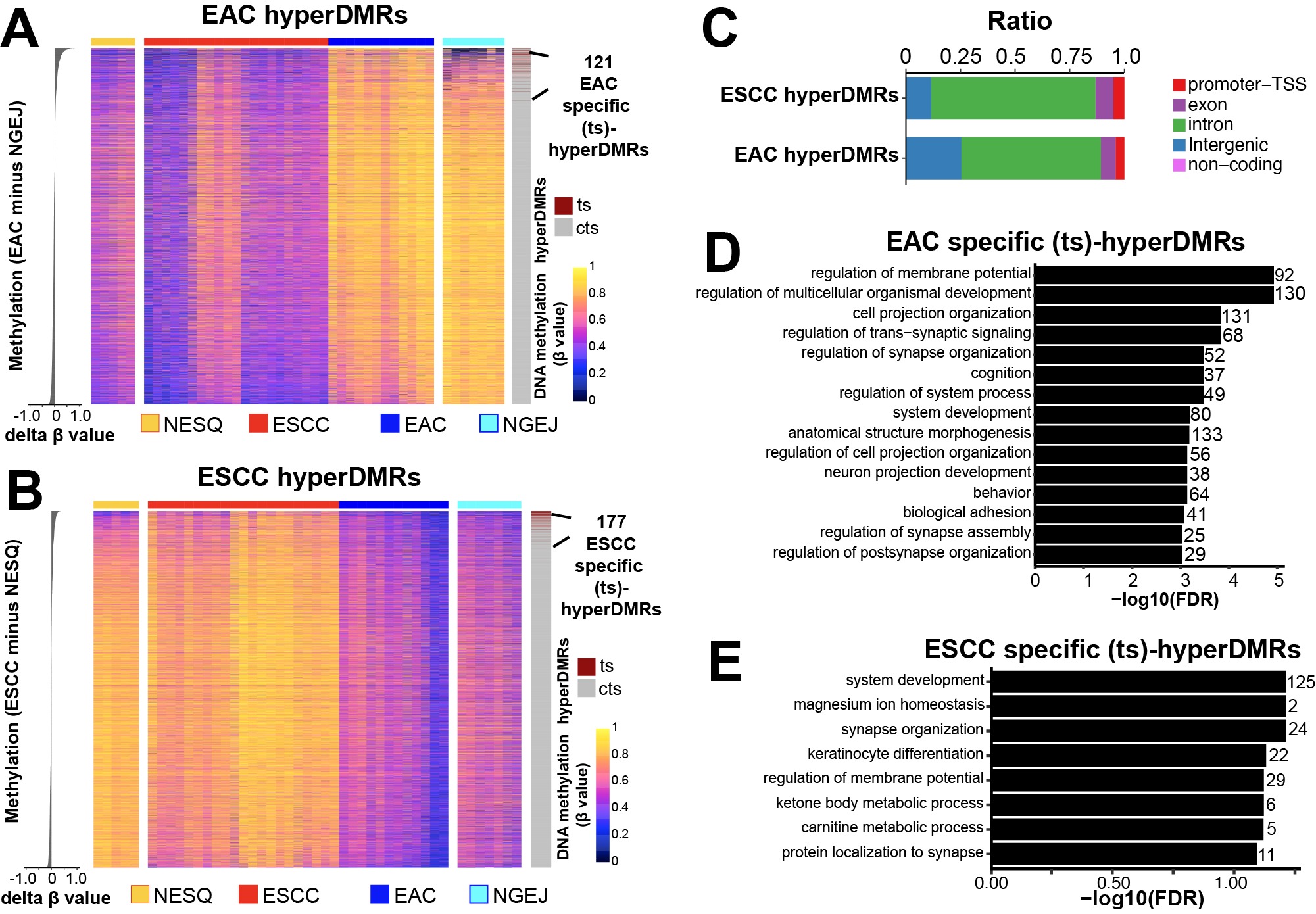


**Figure S6. Characterization of tumor-specific hyperDMRs. (A-B)** Heatmaps showing DNA methylation levels for hyperDMRs in **(A)** EAC or **(B)** ESCC samples. Each column denotes one sample; each row denotes one hyperDMR region. Rows were ordered by delta mean methylation between tumors and corresponding nonmalignant samples. Tumor-specific hypoDMRs were defined using a one-tailed t test between tumors and corresponding nonmalignant samples with the FDR cutoff < 0.05. **(C)** Stacked bar plots showing fractions of ts-hyperDMRs overlapping with different genomic domains. **(D-E)** Cistrome-GO enrichment analyses using downregulated genes in tumors compared with corresponding nonmalignant samples in ts-hyperDMRs from either EAC **(D)** or ESCC **(E)**. Top 15 most significant pathways are shown.


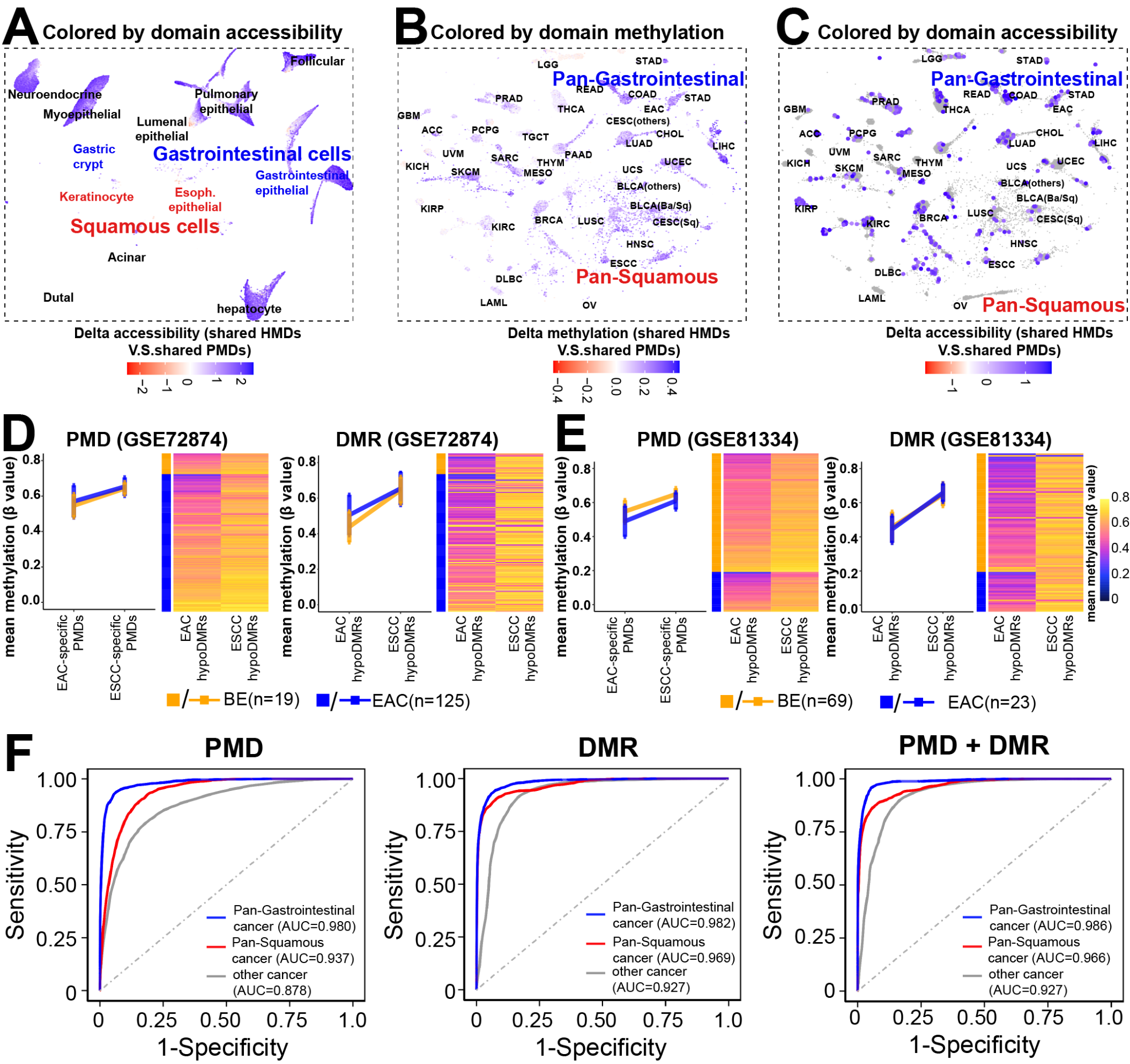


**Figure S7. Neither shared PMDs nor HMDs show cell-type specificity. (A)** UMAP plots showing normal single-cell clusters colored by the delta ATAC-seq accessibility between shared HMDs and PMDs. The total cell number is 145,594. **(B-C)** Pan-cancer clusters are colored by delta methylation **(B)** or ATAC-seq accessibility **(C)** between shared HMDs and PMDs. **(B)** and **(C)** contain 8,915 and 365 tumor samples, respectively. **(D-E)** Line plots and heatmaps respectively showing average and individual methylation levels in BE and EAC samples from two different public datasets. **﻿(F)** ROC curves for three-class classification characterized by the average methylation of subtype-specific PMDs, DMRs or both respectively.
