# Supplementary material for: Comprehensive analyses of partially methylated domains and differentially methylated regions in esophageal cancer reveal both cell-type- and cancer-specific epigenetic regulation
Source: Genome Biol. 2023 Aug 24;24:193. doi: 10.1186/s13059-023-03035-3 (PMC10463844; doi:10.1186/s13059-023-03035-3)
Supplement: Supplementary file 3 — Additional file 3. Review History. [file 13059_2023_3035_MOESM3_ESM.docx]

Review History

**First round of review**

**Reviewer 1**

**Were you able to assess all statistics in the manuscript, including the appropriateness of statistical tests used?**

Yes, and I have assessed the statistics in my report.

**Comments to author:**

In this paper the authors examine the DNA methylation patterns in a series of oesophageal cancer patients, which included the two main types- adenocarcinoma and squamous. They identify differences between these cancer types from their methylation profiles and depending on what type of region they look at PMDs, HMDs, HMRs etc they can see patterns that map to proposed closest normal cells or that distinguish the cancer from the normal state. Using this data, they go on to do some more molecular links and uncover transcriptional regulators that might act in a cancer/cell type-specific way. Overall this is a useful dataset of whole genome methylation which will be of interest to the community working on this cancer. There is also a new tool described, MMSeekR that identifies PMDs and will be of broader use to the DNA methylation community. However, overall the paper is descriptive in nature and does not significantly advance our understanding of the molecular events associated with oesophageal cancer.

Issues to address:

(1) In describing Fig. 1A can the authors justify their choice of NGEJ as the normal cell to compare to EAC? There are multiple proposed cell of origins so this choice needs justifying.

In the text they also mention it being inflamed. Is this therefore not Barrett's tissue?

(2) In fig.2 It is clear that EAC consists of two populations with high and low methylation levels in the blocks shown (the same is apparent in ESCC). Can the authors comment on this with regards to molecular and clinicial insights? If the samples are separated due to this subclassification do we learn anything else from the other analyses shown?

(3) In Fig. 2E, was HiC used in identifying the A and B compartments as this is not clear in the text. If so, which data was used?

(4) In Fig. 3F, SLC2A2 looks to have a very low FDR value. Is this change significant? Also looking at the axes, almost all genes change more than 2 fold (ie log2>1).. This is highly unusual. Can the authors comment on why this is?

(5) Fig. 4A&C need a better colour schemes as the lines are hard to distinguish. Also, can the axes units be standardised between parts A&C so direct comparisons can be made?

(6) In Fig. 5G, why have ETS proteins been grouped as they are in two groups? ETV4/5 do not really resemble ELK1. Would it not be better to call them all ETS-like along with ETS1 etc?

(7) In Fig. 5I and J it would be useful to indicate the % of sites that have GATA motifs in them. Maybe adding an extra column on the left might help.

(8) In Fig. 6 the authors examine hypoDMRs. Can equivalent analyses of hyperDMRs be added?

(9) The finding that HNF4A is likely important in EAC agrees with numerous published studies. The authors suggest and interplay with AP1 which might be novel but no experimental followup to test this hypothesis is provided and it would be recommended to provide such data.

(10) In Fig. 9K-L, why does the model predict things better in all cancers rather than in the training sets which came from oesophageal cancer data.

**Reviewer 2**

**Were you able to assess all statistics in the manuscript, including the appropriateness of statistical tests used?**

There are no statistics in the manuscript.

**Comments to author:**

The paper by Zheng et al. features on a series of extensive analyses on partially methylated domains (PMDs) and differentially methylated regions (DMRs) as co-occuring hallmarks of cancer development. The authors build their initial analyses on two WBGS data sets generated for EAC and ESCC cancers and corresponding normal adjacent tissues but include other data sets at later stages.

The improve the calling of PMDs in cancer cells the authors first introduce a modfied HMM based method by combining MethylSeekR modeling with a multimodal sequence neighborhood based (combined NN + PCC) approach which they name MMSeekR. MMseekR is then shown to better capture PMD's at the extremes allowing an enhanced PMD calling. PMDs in cancers exhibited a high inter-sample heterogeneity in both their depth and extend ranging from 24.3% to 63.4%.

Following the intial PMD calling they perform inter-cancers and and cancer to normal tissues comparisons - showing that a significant proportion of PMD's is cancer (cts) and tissue (ts) specific. A transcriptome analysis supports previous findings (by the authors and others) that cell-type (cancer type) specific PMDs are enriched for cell-type specific genes which tend to be inactive. An interesting finding is that the authors identify a significantly higher somatic mutation rate in EAC-specific PMDs (more extensive) as compared to ESCC-specific PMDs or HMDs (FMRs).

As next steps they define cancer subtype specific DMRs across the genome and subtype-specific DMRs as well as DMRs unique for a given cancer type in relation to the normal tissue and by this defining cancer specific TFs and regulons. For the additional interpretation they use ATAC data as well as ChIP-Seq data (H3K36me2 and H3K27Ac).

A combined analysis of DMRs & PMDs with single-cell results confirmed that both PMDs and hypoDMRs have strong normal cell-type-specificity. The DNA-methylation cell origin signatures strongly suggest that epigenomic changes in PMDs and hypoDMRs occur in normal cells and are maintained in cancer, accompanied by further loss of methylation in PMDs and gain in DMRs. Such region-specific epigenomic features are shared across related cell types.

In their paper the authors present extensive and comprehensive analyses using a combination DMR and PMD calling approaches, multiple datasets and integrating their analyses with other trasncriptomic and epigenomic data. Their findings and observatios are important as they help to better rationalize "old" observations of apparently "antagonistic" methylation changes in cancer, namely how both local and regional (genome wide) hypo- and hypermethylation changes in the genomic landscapes contribute to cancer development. Secondly the paper shows that epigenetic memory imprinted by DNA-methylation allows to dissect cellular origin and cancer specific changes.

The paper is well structured and describes a wealth of data. I have no major concern with the methods and the presentation of results. My minor points are the following:

* MethylSeakR is a standard widely used for PMD calling tool. The method has known limitations to call PMD in cancer cells where extreme changes are occuring (e.g. an HMD to a LMR). Here the 2D/3D approach might help to improve the calling of extreme PMD changes (lines 624-632). However from what is shown in the figures MMSeakR seems to be only marginally improving and mostly call equivalent (Quote: "This 2D model performed comparably well or better than either MethylSeekR or MethPipe in most cases.."). The improvement/contribution by MMSeakR for the entire analysis and particularly to which extend this is compensating cancer sample variation should be outlined in a better way.

* None of the existing DMR callers works perfect. The limitations of the output of called DMRs using Dmrseq package should be discussed.

* The changes in mutation rates (SNV's) is interesting. The association with PMDs could be analysed more specifically: Which base changes predominate? Is there a link to 5mC deamination/repair.

* In various analyses the author work with 450K and EPIC array data (line 198/199 and line 398 following). They do however not clearly state in the text or methods i) on which set and type of data the analyses were done and do not describe ii) how the analyses were performed. Did they infer WGBS methylation calls for PMDs on 450K and EPIC array data and used those for comparisons? A calling of PMDs from 450k/EPIC data is extremely difficult, highly error prone and for 450 K almost impossible.

**Reviewer 3**

**Were you able to assess all statistics in the manuscript, including the appropriateness of statistical tests used?**

Yes, and I have assessed the statistics in my report.

**Comments to author:**

The authors utilise their previous neural network defined score of CpG methylation in combination with the MethylSeekR alpha score to improve the detection of partially methylated domains. These partially methylated domains discovered by their method improve the separation of tumour samples compared to previous state of the art methods, suggesting that the combination of sequence information with methylation values improves the prediction of important regions within the genome.

Further analysis incorporating the histone modification H3K36me2 and ATAC-seq data further suggests reliable PMD localisation.

The authors also note that by masking out of PMDs that this accounts for the global loss of methylation when calling DMRs, suggesting further the value in obtaining these regions.

Minor comments:

1) The authors provide only the raw data for the WGBS human samples. It would be useful to provide at least a bed file of the called PMDs but ideally methylation counts.

2) The MMSeekR package relies on a precomputed neural network score that is provided in the https://github.com/yuanzi2/MMSeekR.data repository. It would be useful to identify how to apply this to other genomes? e.g. mouse?

3) In methods section: Development of a sequence-aware PMD calling method: Multi-model PMD SeekR, the figures referred to are Fig S1 but it looks like they should be S2?

**Authors Response**

**Point-by-point responses to the reviewers’ comments:**

Reviewer #1:

In this paper the authors examine the DNA methylation patterns in a series of oesophageal cancer patients, which included the two main types- adenocarcinoma and squamous. They identify differences between these cancer types from their methylation profiles and depending on what type of region they look at PMDs, HMDs, HMRs etc they can see patterns that map to proposed closest normal cells or that distinguish the cancer from the normal state. Using this data, they go on to do some more molecular links and uncover transcriptional regulators that might act in a cancer/cell type-specific way. Overall this is a useful dataset of whole genome methylation which will be of interest to the community working on this cancer. There is also a new tool described, MMSeekR that identifies PMDs and will be of broader use to the DNA methylation community. However, overall the paper is descriptive in nature and does not significantly advance our understanding of the molecular events associated with oesophageal cancer.

*Authors' Response: We are truly appreciative of the Reviewer’s careful evaluation of the work and, we thank you for these valuable comments and suggestions below!*

Issues to address:

(1) In describing Fig. 1A can the authors justify their choice of NGEJ as the normal cell to compare to EAC? There are multiple proposed cell of origins so this choice needs justifying.

In the text they also mention it being inflamed. Is this therefore not Barrett's tissue?

*Authors' Response: The Reviewer is correct that the cell-of-origin for EAC has been under intense investigation, which has yet to reach a final consensus. Nevertheless, the most recent study published in Science used single-cell genomics to identify NGEJ or cardia cells as the most likely cell-of-origin of EAC[1]. In humans, NGEJ and gastric cardia tissues are anatomically and surgically indistinguishable[2]; indeed, both NGEJ and gastric cardia samples have been used as nonmalignant controls for EAC in multiple molecular studies[1,3,4].*

*We have examined these NGEJ samples pathologically and histologically, which contained limited infiltration of lymphocytes but showed no evidence of Barrett's esophagus (i.e., no goblet cells).*

*We have now added justifications of using NGEJ cells as nonmalignant controls for EAC in the revised manuscript (Page 4, underlined).*

*(2) In fig.2 It is clear that EAC consists of two populations with high and low methylation levels in the blocks shown (the same is apparent in ESCC). Can the authors comment on this with regards to molecular and clinicial insights? If the samples are separated due to this subclassification do we learn anything else from the other analyses shown?*

*Authors' Response: Indeed, in our analyses both ESCC and EAC tumors exhibited strong variation at their PMD methylation levels. This is consistent with recent publications reporting high inter-tumor methylation heterogeneity in both ESCC[5] and EAC samples[3,6,7]. Moreover, we and others have reported considerable variations in the degree of loss within large PMD blocks in other cancer types[8,9]. While not well understood, the variation is hypothesized to arise from a combination of factors including the composition of stromal cells and proliferative rate of cancer cells, but can also be modified by other epigenetic changes. Unfortunately, since we did not have matched DNA-sequencing or RNA-sequencing data of the present cohort, it is difficult to determine other molecular changes associated with these DNA methylation profiles.*

*The Reviewer also asked if there were potential associations between methylation features and clinicopathological parameters. While the size of our cohort is statistically underpowered for such association analyses, we addressed this using published large-scale datasets, such as The Cancer Genome Atlas (TCGA, containing 90 ESCC samples and 87 EAC samples, profiled using the HM450K methylation array platform[10]). Specifically, we correlated the methylation levels of subtype-specific PMD blocks, to each of known risk factors and clinicopathological parameters for these cancer types. We found that none of these factors, including age, smoking history, alcohol consumption, lymph node metastasis, clinical stage, has significant impact on either subtype-specific PMD blocks (Additional file 1: Fig. S3H; Page7 second paragraph).*

*To further validate these results, we analyzed additional whole genome bisulfite sequencing (WGBS) data from PRJNA523898 (ESCC=42)[11]. Importantly, these independent samples again confirmed that there was no association between ESCC-specific PMDs with either age, clinical stage or lymph node metastasis (Additional file 1: Fig. S3I; Page7 second paragraph).*

(3) In Fig. 2E, was HiC used in identifying the A and B compartments as this is not clear in the text. If so, which data was used?

*Authors' Response: In Fig. 2E, we reconstructed chromatin A/B compartments using the minfi compartments function[12] based on the HM450K methylation data from the TCGA esophageal cancer dataset[10]. The minfi compartments method is based on the finding that A/B compartments can be reliably estimated from the HM450K methylation microarray primarily using the pairwise correlation among DNA methylation beta values. Importantly, there is a 93% agreement of the estimation of A/B compartments between HiC and HM450K methods.*

*We have now added the description of how we used this method in the Result and Method sections (Page 18, underlined), which is pasted below for your review:*

*“HM450K methylation data from the TCGA were used to estimate the chromatin A/B compartments using minfi compartments function with “resolution=100*1000, what=OpenSea” options. Briefly, ~170,000 open sea probes on the HM450k array showed the strongest correlation with A/B compartments and were used in the prediction process. A p x n methylation matrix was generated for each chromosome, where p refers to the normalized probes and n represents the samples. Next, we calculated the correlation between pairwise probes and obtained the p x p correlation matrix. Then the correlation matrix was grouped into bins based on a predetermined resolution k and the median correlation between the CpGs contained in each bin was calculated. Bins without any probes were removed.”*

(4) In Fig. 3F, SLC2A2 looks to have a very low FDR value. Is this change significant? Also looking at the axes, almost all genes change more than 2 fold (ie log2>1). This is highly unusual. Can the authors comment on why this is?

*Authors' Response: The FDR of SLC2A2 is 3.7e-15 and the fold change is -3.47 when comparing ESCC vs. EAC. While this FDR value is very highly significant, it appears modest in the volcano plot due to the wide range of the Y axis.*

*The Reviewer also commented that a large number of genes showed fold change > 2. In our differential expression analysis, we included the entire annotated genes in the GENCODE database (n=60,483), regardless of their baseline expression levels. As a result, 10,663 and 6,866 genes were significantly upregulated in ESCC and EAC, respectively (FDR<0.01, fold change>2). The visual impression that most of the genes have fold change log2>1 is caused by the scale of the axes that accommodate outliers with very large -log10 FDR values (up to 200) and very large log2 fold changes (up to 10). Therefore, while the majority of genes (71%) do not change their expression (log2<1), they are compressed into a tiny space in the center-bottom of the plot.*

*Taking into account the Reviewer’s comment, now we have removed those non-expressed genes, defined as average expression level (FPKM)<0.1, and obtained 3,432 upregulated genes in ESCC and 3,564 in EAC. We have updated this figure and the related method accordingly (Revised Main Fig. 3F; Page 21, underlined).*

(5) Fig. 4A&C need a better colour schemes as the lines are hard to distinguish. Also, can the axes units be standardised between parts A&C so direct comparisons can be made?

*Authors' Response: As suggested, we have revised the color schemes to better distinguish different groups. We have also unified the scale of Y axes between panel A and C so they can be compared directly (Revised Main Fig. 4A and 4C).*

(6) In Fig. 5G, why have ETS proteins been grouped as they are in two groups? ETV4/5 do not really resemble ELK1. Would it not be better to call them all ETS-like along with ETS1 etc?

*Authors' Response: We downloaded the annotation of the TF family and subfamily from the TFClass database[13]. While ELK1, ETV4/5, ETS1 and GABPA are all from the Ets-related factors family, ELK1 and ETV4/5 belong to the Elk-like factors subfamily, and ETS1 and GABPA belong to the Ets-like factors subfamily.*

*Nevertheless, we agree with the Reviewer's comment and we have now revised this figure by grouping TFs using their family-level assignment (Revised Main Fig. 5G-H).*

(7) In Fig. 5I and J it would be useful to indicate the % of sites that have GATA motifs in them. Maybe adding an extra column on the left might help.

*Authors' Response: We agree and this is a really good suggestion. We have revised these two panels accordingly, by adding the proportion of sites with motifs of GATA4 (54.5%) and TP63 (81.1%), using pie charts at the upper left corners (Revised Main Fig. 5I-J). We have also updated the text in the revised manuscript (Page 10, underlined).*

(8) In Fig. 6 the authors examine hypoDMRs. Can equivalent analyses of hyperDMRs be added?

*Authors' Response: As suggested, we performed equivalent analyses to identify tumor-specific (ts)-hyperDMRs (Revised Additional file 1: Fig. S6A-C). Compared with ts-hypoDMRs (n=1,972 for EAC and n=654 for ESCC, Main Fig. 6A, Additional file 1: Fig. S5A), there are much fewer ts-hyperDMRs (n=121 for EAC and n=177 for ESCC). With respect to functional enrichment of genes within ts-hypoDMRs, our original submission showed that they were strongly enriched in cancer related pathways such as cell cycle transition (in both EAC and ESCC, Main Fig. 6C-D). In contrast, here we did not observe cancer-related pathways enriched in ts-hyperDMRs (Revised Additional file 1: Fig. S6D-E), indicating that ts-hyperDMRs may not contain genes implicated in cancer biology. We also note that the -log10 FDRs of the ts-hyperDMRs have much lower statistical significance than the ts-hypoDMRs, as would be expected from the small number.*

*Nevertheless, we still incorporated these new analyses into the revised manuscript as Additional file 1: Fig. S6 with description on Page 11 (underlined), in order to provide more comprehensive information for the readers.*

(9) The finding that HNF4A is likely important in EAC agrees with numerous published studies. The authors suggest and interplay with AP1 which might be novel but no experimental followup to test this hypothesis is provided and it would be recommended to provide such data.

*Authors' Response: We agree with the Reviewer’s recommendation that experimental validation would strengthen our findings and increase the novelty of our work.*

*Our initial submission performed TF motif sequence enrichment analysis which indicated that AP1 factors (e.g., FOSL1, FOSL2, JUN) might cooperatively occupy EAC tumor-specific DMRs together with HNF4A. To address this, we utilized ESO26 and OE19 cells (EAC cell lines) from which we and others previously published HNF4A ChIP-Seq data[14,15]. We first intersected EAC tumor-specific DMRs with shared HNF4A-binding peaks from these two cell lines, identifying 160 HNF4A-occupying tumor-specific DMRs.*

*The AP1 family has multiple members[16], and in this experimental validation we focused on the FOSL1 gene, since it is the only highly overexpressed AP1 factor in EAC tumors vs. nonmalignant samples (Main Fig. 6E). Importantly, we found that 58 of the 160 HNF4A-occupying tumor-specific DMRs also contained FOSL1 recognition motif sequence, again confirming our initial observation that FOSL1 motif significantly co-occurs with HNF4A motif in EAC tumor-specific DMRs (Main Fig. 6H).*

*To functionally test whether HNF4A occupies these 58 tumor-specific DMRs cooperatively with FOSL1, we randomly selected 10 of these 58 regions for ChIP-qPCR assay using a validated FOSL1 ChIP antibody[17,18]. Moreover, we performed HNF4A knockdown (Revised Main Fig.6I) to compare the occupancy of FOSL1 in the presence vs. absence of HNF4A binding. We first validated that FOSL1 binds to 7 and 6 out of these 10 regions in ESO26 and OE19 cells, respectively. Importantly, upon the knockdown of HNF4A, the binding of FOSL1 was significantly reduced in 5 out of the 7 regions in ESO26 cells (with the remaining two showing the same trend without statistical significance), and 3 out of 6 regions in OE19 cells (with an additional 2 showing the same trend without statistical significance) (Revised Main Fig.6J-K). These results suggest that HNF4A is functionally required for the occupancy of FOSL1 on a subset of EAC tumor-specific DMRs, a possible mechanism underlying the strong co-occurrence of their motif sequences in these epigenetic regions.*

*We have added these new results into the revised manuscript (Revised Main Fig. 6I-K; Page 11, underlined).*

(10) In Fig. 9K-L, why does the model predict things better in all cancers rather than in the training sets which came from oesophageal cancer data.

*Authors' Response: The Reviewer raised a valid concern. We carefully checked our classification model and realized that the sample numbers for each group were unbalanced: there were 875 GI cancers, 1,370 squamous cancers, but 6,670 other cancers. Because we used a multinomial regression model and the other cancers made up significantly more than half of the entire samples, this resulted in a high performance for “other cancers”.*

*To address this, we performed sample downsizing and randomly selected 20% samples of other cancers to achieve a balanced training set. We re-applied the multinomial logistic regression model for data training. This process was repeated 100 times and the training results were merged. Indeed, as shown in Revised Fig. 8K-L, both the PMDs and DMRs now have higher predictive performance on GI and squamous cancers than other cancers. We have updated these results (Page 13, underlined) and methods (Page 22-23, underlined) in the revised manuscript.*

*We thank the Reviewer for your very careful review and raising of this important point.*

Reviewer #2:

The paper by Zheng et al. features on a series of extensive analyses on partially methylated domains (PMDs) and differentially methylated regions (DMRs) as co-occurring hallmarks of cancer development. The authors build their initial analyses on two WBGS data sets generated for EAC and ESCC cancers and corresponding normal adjacent tissues but include other data sets at later stages.

To improve the calling of PMDs in cancer cells the authors first introduce a modified HMM based method by combining MethylSeekR modeling with a multimodal sequence neighborhood based (combined NN + PCC) approach which they name MMSeekR. MMseekR is then shown to better capture PMD's at the extremes allowing an enhanced PMD calling. PMDs in cancers exhibited a high inter-sample heterogeneity in both their depth and extend ranging from 24.3% to 63.4%.

Following the initial PMD calling they perform inter-cancers and cancer to normal tissues comparisons - showing that a significant proportion of PMD's is cancer (cts) and tissue (ts) specific. A transcriptome analysis supports previous findings (by the authors and others) that cell-type (cancer type) specific PMDs are enriched for cell-type specific genes which tend to be inactive. An interesting finding is that the authors identify a significantly higher somatic mutation rate in EAC-specific PMDs (more extensive) as compared to ESCC-specific PMDs or HMDs (FMRs).

As next steps they define cancer subtype specific DMRs across the genome and subtype-specific DMRs as well as DMRs unique for a given cancer type in relation to the normal tissue and by this defining cancer specific TFs and regulons. For the additional interpretation they use ATAC data as well as ChIP-Seq data (H3K36me2 and H3K27Ac).

A combined analysis of DMRs & PMDs with single-cell results confirmed that both PMDs and hypoDMRs have strong normal cell-type-specificity. The DNA-methylation cell origin signatures strongly suggest that epigenomic changes in PMDs and hypoDMRs occur in normal cells and are maintained in cancer, accompanied by further loss of methylation in PMDs and gain in DMRs. Such region-specific epigenomic features are shared across related cell types.

In their paper the authors present extensive and comprehensive analyses using a combination DMR and PMD calling approaches, multiple datasets and integrating their analyses with other transcriptomic and epigenomic data. Their findings and observations are important as they help to better rationalize "old" observations of apparently "antagonistic" methylation changes in cancer, namely how both local and regional (genome wide) hypo- and hypermethylation changes in the genomic landscapes contribute to cancer development. Secondly the paper shows that epigenetic memory imprinted by DNA-methylation allows to dissect cellular origin and cancer specific changes.

The paper is well structured and describes a wealth of data. I have no major concern with the methods and the presentation of results. My minor points are the following:

*Authors' Response: We are abundantly grateful for the Reviewer’s meticulous evaluation of our manuscript, and are very delighted that the Reviewer remarks that our work is well structured and describes a wealth of data.*

*We also thank the Reviewer for the valuable comments, which we have addressed point-by-point below.*

(1) MethylSeakR is a standard widely used for PMD calling tool. The method has known limitations to call PMD in cancer cells where extreme changes are occuring (e.g. an HMD to a LMR). Here the 2D/3D approach might help to improve the calling of extreme PMD changes (lines 624-632). However from what is shown in the figures MMSeakR seems to be only marginally improving and mostly call equivalent (Quote: "This 2D model performed comparably well or better than either MethylSeekR or MethPipe in most cases.."). The improvement/contribution by MMSeakR for the entire analysis and particularly to which extend this is compensating cancer sample variation should be outlined in a better way.

*Authors' Response: Thank you for your comment! Our initial submission did show the practical benefit of MMSeekR by presenting the improved clustering of cell types (Main Fig. 1D, Additional file 1: Fig. S2G), as well as PMD calling in each sample (Additional file 1: Fig. S2E, I). Nonetheless, we agree with the Reviewer that it will be very helpful to compare the performance of our method (MMSeekR) vs. MethylSeakR and MethPipe in a more quantitative and systematic manner, in order to provide the readers and future users with more information for consideration of the use of these methods.*

*To address this, we performed a Precision-Recall analysis and used the common PMDs (defined as shared PMD regions across 40 different cancer types)[9] as the reference for true positives. For each sample, we calculated the F1-score (a harmonic measure of both precision and recall scores) using our esophageal cohort (two tumor types and two normal types, total 45 samples) and the Blueprint tumor cohort (6 tumor types, total 49 samples), which are the same set of samples included in the initial submission.*

*Importantly, this analysis showed that the F1 score of MMSeekR was the highest in almost every group comparison (Revised Additional file 1: Fig. S2F and S2J). While the score was sometimes only marginally better in MMSeekR, it was more consistent than MethylSeekR, which performed quite poorly on multiple cancer types (e.g., ALL, MM, AML). Overall, MMSeekR had an average of F1 = 0.715 whereas MethylSeekR had an average of F1= 0.689.*

*In our initial submission, we performed Principal Component Analysis (PCA) using PMDs identified by the three different methods, which showed that MMSeekR outperformed MethylSeekR and MethPipe, completely separating the samples from different cancer types (Main Fig. 1D and Additional file 1: Fig. S2G). During the revision, we further quantified this by computing the average distance in PCA space between tumors of the same subtype (intra-tumor) and between tumors across both subtypes (inter-tumor), taking the inter-tumor to intra-tumor ratio (Revised Additional file 1: Fig. S2H and S2K). This analysis further confirmed our initial findings since MMSeekR always had the highest ratio.*

*We have added these new results into the revised manuscript (Page 5, underlined; Additional file 1: Fig. S2F, H, J, K).*

(2) None of the existing DMR callers works perfect. The limitations of the output of called DMRs using Dmrseq package should be discussed.

*Authors' Response: Compared to DMRcate, DSS and other DMR callers[19–21], DMRseq detects DMRs from WGBS data in a more comprehensive manner, via: i) discovering candidate regions, ii) scoring candidate regions with an exchangeable (across the genome) statistic and evaluating statistical significance using a random permutation test on the pooled null distribution of scores. This method improves the specificity and sensitivity of DMR calling and accurately controls the false discovery rate. DMRseq’s performance further improves and becomes more consistent as the “cutoff” and “maxPerms” increase. Here, the “cutoff” is a screening threshold denoting the single CpG coefficient that is used to discover candidate regions. The “maxPerms” is a positive integer representing the maximum number of permutations that are used to generate the global null distribution.*

*However, these improvements come at the expense of CPU time. In particular, the maxPerms function is time-consuming. We found that a default maxPerms value of 10 (i.e., 10 permutations) produced very low reproducibility and we increased it to 500 to obtain accurate, robust and consistent DMR results. However, compared to the default setting (which takes between 2~12 minutes for each chromosome when comparing 21 ESCC vs 12 EAC samples using one CPU), it takes more than 2-5 hours when the maxPerms value is increased to 500. The other limitation of DMRseq is that some of the DMR regions identified can be very long (e.g., more than 20% DMRs are over 5kb), which may overlap with PMD regions. Therefore, in order to factor out the effect of PMD hypomethylation, we masked any PMD regions during DMR calling.*

*We have added these discussions briefly in the revised manuscript (Page 20, underlined).*

(3) The changes in mutation rates (SNV's) is interesting. The association with PMDs could be analysed more specifically: Which base changes predominate? Is there a link to 5mC deamination/repair.

*Authors' Response: The Reviewer raised a very interesting question. To address this comprehensively, we investigated the mutational signatures using the method MutationalPatterns[22], which has been widely used to determine the contribution of established (i.e., the COSMIC 49 single-base-substitution (SBS) signatures[23]) or user-specified mutational signatures in individual samples. We first validated our results by analyzing genome-wide somatic mutations, and confirmed that both ESCC and EAC tumors are associated with SBS1 (5mC deamination), SBS40 (age-related), SBS5 (age-related) (Revised Additional file 1: Fig.S3G). We also confirmed cancer-specific signatures, including SBS17b in EAC (possibly associated with gastric acid reflux[24]), and SBS2 and SBS13 in ESCC (APOBEC induced).*

*We next similarly identified mutational signatures using somatic mutations only within cancer-specific PMD regions. While most of the mutational signatures had comparable weight between genome-wide mutations and PMD-restricted mutations, we noted a consistent and conspicuous decrease of SBS40 and SBS5 signatures in cancer-specific PMDs in both ESCC and EAC tumors. We find this result interesting and supportive of our findings on PMDs: since cancer-specific PMDs occur during tumor development, which is independent of age. Therefore, mutations within cancer-specific PMDs display much lower age-related signatures than genome-wide mutations.*

*We have incorporated these new data into the revised manuscript as Additional file 1: Fig.S3G, with description on Page 6-7 (underlined).*

(4) In various analyses the author work with 450K and EPIC array data (line 198/199 and line 398 following). They do however not clearly state in the text or methods i) on which set and type of data the analyses were done and do not describe ii) how the analyses were performed. Did they infer WGBS methylation calls for PMDs on 450K and EPIC array data and used those for comparisons? A calling of PMDs from 450k/EPIC data is extremely difficult, highly error prone and for 450 K almost impossible.

*Authors' Response: We understand the Reviewer’s confusion and we apologize for not describing the information more clearly in the manuscript.*

*Indeed, PMD identification using 450K/EPIC-array data is highly challenging, inaccurate and error prone. Therefore, we did not call PMD from any 450K/EPIC-array data. Instead, we identified PMD regions only from our WGBS dataset. We then inferred DNA methylation values of PMDs in 450K/EPIC-array data by using PMD-overlapped probes from 450K/EPIC arrays. This analysis was used in Main Fig. 2D, Fig. 8B-E, K-M and Additional file 1: Fig. S3D, and Fig. S7B, D-F.*

*Regarding the source of the 450K-array data, they were from the TCGA esophageal cancer project, containing 91 ESCC and 75 EAC samples[25]. The EPIC array data were obtained from the Oesophageal Cancer Clinical and Molecular Stratification (OCCAMS) consortium (EGAD00010001822), containing 292 EAC samples[3]. With respect to the array data analysis, the methylation value of each probe was extracted using the SeSAME package with noob and dyeBiasCorrTypeINorm function for background subtraction and dye bias correction[26].*

*We have now described the data source and data analysis clearly in the revised manuscript (Page 17-18; Figure legends, underlined).*

*We thank the Reviewer again for these critical comments!*

Reviewer #3:

The authors utilise their previous neural network defined score of CpG methylation in combination with the MethylSeekR alpha score to improve the detection of partially methylated domains. These partially methylated domains discovered by their method improve the separation of tumour samples compared to previous state of the art methods, suggesting that the combination of sequence information with methylation values improves the prediction of important regions within the genome.

Further analysis incorporating the histone modification H3K36me2 and ATAC-seq data further suggests reliable PMD localisation.

The authors also note that by masking out of PMDs that this accounts for the global loss of methylation when calling DMRs, suggesting further the value in obtaining these regions.

Minor comments:

(1) The authors provide only the raw data for the WGBS human samples. It would be useful to provide at least a bed file of the called PMDs but ideally methylation counts.

*Authors' Response: Thank you for reviewing and commenting on our work!*

*As suggested by the Reviewer, we have now provided a bed file of DNA methylation counts, and uploaded to Zenodo, which is publicly accessible (https://zenodo.org/record/6954946#.ZFuKUuxByWY). This information has been added to the revised manuscript (Page 23, underlined).*

(2) The MMSeekR package relies on a precomputed neural network score that is provided in the https://urldefense.com/v3/__https://github.com/yuanzi2/MMSeekR.data__;!!LIr3w8kk_Xxm!vP1HHNOupNCzvEeJRaRjmMmTf0ldgHDTwwY_CcGCEKWc0c9TERekiaE2GrXtv65Ch11UuZ1CmljyV4A$ repository. It would be useful to identify how to apply this to other genomes? e.g. mouse?

*Authors' Response: Yes, we also believe that it would be useful if neural network-based score systems can be applied to other genomes. In fact, this is what we have been working on for some time. However, since training the neural network program requires a large amount of WGBS data, the progress in other model genomes (such as mouse) has been slower than human’s.*

(3) In methods section: Development of a sequence-aware PMD calling method: Multi-model PMD SeekR, the figures referred to are Fig S1 but it looks like they should be S2?

*Authors' Response: Yes, thank you for spotting this typo, which has been corrected (Page 19, underlined).*

*References*

*1. Nowicki-Osuch K, Zhuang L, Jammula S, Bleaney CW, Mahbubani KT, Devonshire G, et al. Molecular phenotyping reveals the identity of Barrett’s esophagus and its malignant transition. Science. 2021;373:760–7.*

*2. Quante M, Wang TC, Bass AJ. Adenocarcinoma of the oesophagus: is it gastric cancer? Gut [Internet]. BMJ Publishing Group; 2022 [cited 2023 Apr 25]; Available from: https://gut.bmj.com/content/early/2022/03/31/gutjnl-2022-327096.abstract*

*3. Jammula S, Katz-Summercorn AC, Li X, Linossi C, Smyth E, Killcoyne S, et al. Identification of Subtypes of Barrett’s Esophagus and Esophageal Adenocarcinoma Based on DNA Methylation Profiles and Integration of Transcriptome and Genome Data. Gastroenterology [Internet]. Gastroenterology; 2020 [cited 2023 Apr 25];158. Available from: https://pubmed.ncbi.nlm.nih.gov/32032585/*

*4. Chen L, Huang M, Plummer J, Pan J, Jiang YY, Yang Q, et al. Master transcription factors form interconnected circuitry and orchestrate transcriptional networks in oesophageal adenocarcinoma. Gut. 2020;69:630–40.*

*5. Liu Z, Zhao Y, Kong P, Liu Y, Huang J, Xu E, et al. Integrated multi-omics profiling yields a clinically relevant molecular classification for esophageal squamous cell carcinoma. Cancer Cell. 2023;41:181–95.e9.*

*6. Yu M, Maden S, Stachler M, Kaz AM, Ayers J, Guo Y, et al. Subtypes of Barrett’s Esophagus and Esophageal Adenocarcinoma Based on Genome-wide Methylation Analysis. Gut. NIH Public Access; 2019;68:389.*

*7. Krause L, Nones K, Loffler KA, Nancarrow D, Oey H, Tang YH, et al. Identification of the CIMP-like subtype and aberrant methylation of members of the chromosomal segregation and spindle assembly pathways in esophageal adenocarcinoma. Carcinogenesis [Internet]. Carcinogenesis; 2016 [cited 2023 Apr 25];37. Available from: https://pubmed.ncbi.nlm.nih.gov/26905591/*

*8. Brinkman AB, Nik-Zainal S, Simmer F, Rodríguez-González FG, Smid M, Alexandrov LB, et al. Partially methylated domains are hypervariable in breast cancer and fuel widespread CpG island hypermethylation. Nat Commun. 2019;10:1749.*

*9. Zhou W, Dinh HQ, Ramjan Z, Weisenberger DJ, Nicolet CM, Shen H, et al. DNA methylation loss in late-replicating domains is linked to mitotic cell division. Nat Genet. 2018;50:591–602.*

*10. Li X, Ma W, Liu H, Wang D, Su L, Yang X. Integrative pan-cancer analysis of cuproplasia-associated genes for the genomic and clinical characterization of 33 tumors. Chin Med J [Internet]. 2023; Available from: http://dx.doi.org/10.1097/CM9.0000000000002343*

*11. Teng H, Xue M, Liang J, Wang X, Wang L, Wei W, et al. Inter- and intratumor DNA methylation heterogeneity associated with lymph node metastasis and prognosis of esophageal squamous cell carcinoma. Theranostics. 2020;10:3035–48.*

*12. Fortin J-P, Hansen KD. Reconstructing A/B compartments as revealed by Hi-C using long-range correlations in epigenetic data. Genome Biol. BioMed Central; 2015;16:1–23.*

*13. Wingender E, Schoeps T, Haubrock M, Krull M, Dönitz J. TFClass: expanding the classification of human transcription factors to their mammalian orthologs. Nucleic Acids Res. 2018;46:D343–7.*

*14. Pan J, Silva TC, Gull N, Yang Q, Plummer JT, Chen S, et al. Lineage-Specific Epigenomic and Genomic Activation of Oncogene HNF4A Promotes Gastrointestinal Adenocarcinomas. Cancer Res. 2020;80:2722–36.*

*15. Rogerson C, Britton E, Withey S, Hanley N, Ang YS, Sharrocks AD. Identification of a primitive intestinal transcription factor network shared between esophageal adenocarcinoma and its precancerous precursor state. Genome Res. 2019;29:723–36.*

*16. Shaulian E, Karin M. AP-1 as a regulator of cell life and death. Nat Cell Biol [Internet]. Nat Cell Biol; 2002 [cited 2023 May 4];4. Available from: https://pubmed.ncbi.nlm.nih.gov/11988758/*

*17. Zhang M, Hoyle RG, Ma Z, Sun B, Cai W, Cai H, et al. FOSL1 promotes metastasis of head and neck squamous cell carcinoma through super-enhancer-driven transcription program. Mol Ther. 2021;29:2583–600.*

*18. Guneri-Sozeri PY, Özden-Yılmaz G, Kisim A, Cakiroglu E, Eray A, Uzuner H, et al. FLI1 and FRA1 transcription factors drive the transcriptional regulatory networks characterizing muscle invasive bladder cancer. Commun Biol. 2023;6:199.*

*19. Peters TJ, Buckley MJ, Chen Y, Smyth GK, Goodnow CC, Clark SJ. Calling differentially methylated regions from whole genome bisulphite sequencing with DMRcate. Nucleic Acids Res. 2021;49:e109.*

*20. Park Y, Wu H. Differential methylation analysis for BS-seq data under general experimental design. Bioinformatics. 2016;32:1446–53.*

*21. Dolzhenko E, Smith AD. Using beta-binomial regression for high-precision differential methylation analysis in multifactor whole-genome bisulfite sequencing experiments. BMC Bioinformatics. 2014;15:215.*

*22. Blokzijl F, Janssen R, van Boxtel R, Cuppen E. MutationalPatterns: comprehensive genome-wide analysis of mutational processes. Genome Med. BioMed Central; 2018;10:1–11.*

*23. Alexandrov LB, Kim J, Haradhvala NJ, Huang MN, Tian Ng AW, Wu Y, et al. The repertoire of mutational signatures in human cancer. Nature. Nature Publishing Group; 2020;578:94–101.*

*24. Lin D-C, Dinh HQ, Xie J-J, Mayakonda A, Silva TC, Jiang Y-Y, et al. Identification of distinct mutational patterns and new driver genes in oesophageal squamous cell carcinomas and adenocarcinomas. Gut. BMJ Publishing Group; 2018;67:1769–79.*

*25. Integrated genomic characterization of oesophageal carcinoma. Nature [Internet]. Nature; 2017 [cited 2023 May 10];541. Available from: https://pubmed.ncbi.nlm.nih.gov/28052061/*

*26. Zhou W, Triche TJ Jr, Laird PW, Shen H. SeSAMe: reducing artifactual detection of DNA methylation by Infinium BeadChips in genomic deletions. Nucleic Acids Res. 2018;46:e123.*

**Second round of review**

**Reviewer 1**

Generally the authors have responded well to my comments. However, while the authors have added some limited functional data to show an interplay between HNF4A and AP1, fundamentally the findings are rather confirmatory still, as both factors have previously been associated with EAC.

In its current form, the abstract would need changing to indicate that the HNF4A-AP1 interplay only applies to EAC as this is currently not clearly stated. Also the results text should be altered to say that a subset of FOSL2 bound regions show reduced binding following HNF4A treatment/ "majority" is a bit of an overstatement, especially for OE19 cells. Also, the number of biological replicates should be added to the figure legend.

**Reviewer 2**

no further comments and questions.

**Authors Response**

**Point-by-point responses to the reviewers’ comments:**

*We have revised our manuscript accordingly, and addressed the remaining comments from Referee 1 as well as editorial requirements. To ensure transparency, all changes have been highlighted by underline in the revised manuscript.*
